# Regulation of peripheral Th/Treg differentiation and suppression of airway inflammation by Nr4a transcription factors

**DOI:** 10.1016/j.isci.2021.102166

**Published:** 2021-02-07

**Authors:** Takashi Sekiya, Shizuko Kagawa, Katsunori Masaki, Koichi Fukunaga, Akihiko Yoshimura, Satoshi Takaki

**Affiliations:** 1 Section of Immune Response Modification, Department of Immune Regulation, The Research Center for Hepatitis and Immunology, National Center for Global Health and Medicine, 1-7-1 Kohnodai, Ichikawa, Chiba 272-8516, Japan; 2 Department of Immune Regulation, The Research Center for Hepatitis and Immunology, National Center for Global Health and Medicine, 1-7-1 Kohnodai, Ichikawa, Chiba 272-8516, Japan; 3 Department of Microbiology and Immunology, Keio University School of Medicine, 35 Shinanomachi, Shinjuku-ku, Tokyo 160-8582, Japan; 4 Division of Pulmonary Medicine, Department of Medicine, Keio University School of Medicine, Tokyo 160-8582, Japan

**Keywords:** Biological Sciences, Immunology, Cell Biology

## Abstract

Helper T (Th) and regulatory T (Treg) cell differentiation programs promote the eradication of pathogens, while minimizing adverse immune reactions. Here, we found that Nr4a family of nuclear receptors supports Treg cell induction and represses Th1 and Th2 cell differentiation from naive CD4^+^ T cells. Nr4a factors are transiently induced in CD4^+^ T cells immediately after antigen stimulation, thereby mediating epigenetic changes. In differentiating Treg cells, Nr4a factors mainly upregulated the early responsive genes in the Treg cell-specifying gene set, either directly or in cooperation with Ets family transcription factors. In contrast, Nr4a factors repressed AP-1 activity by interrupting a positive feedback loop for Batf factor expression, thus suppressing Th2 cell-associated genes. In an allergic airway inflammation model, Nr4a factors suppressed the pathogenesis, mediating oral tolerance. Lastly, pharmacological activation of an engineered Nr4a molecule prevented allergic airway inflammation, indicating that Nr4a factors may be novel therapeutic targets for inflammatory diseases.

## Highlights

• Among “Treg signature genes”, Nr4a factors mainly induce early responsive ones
• Nr4a activate target genes directly or by supporting Ets factors' function
• Nr4a factors repress Th2-driving positive feedback loop for Batf factor expression
• Pharmacological activation of Nr4a factors' activity prevented airway inflammation

## Graphical abstract

**Figure:**
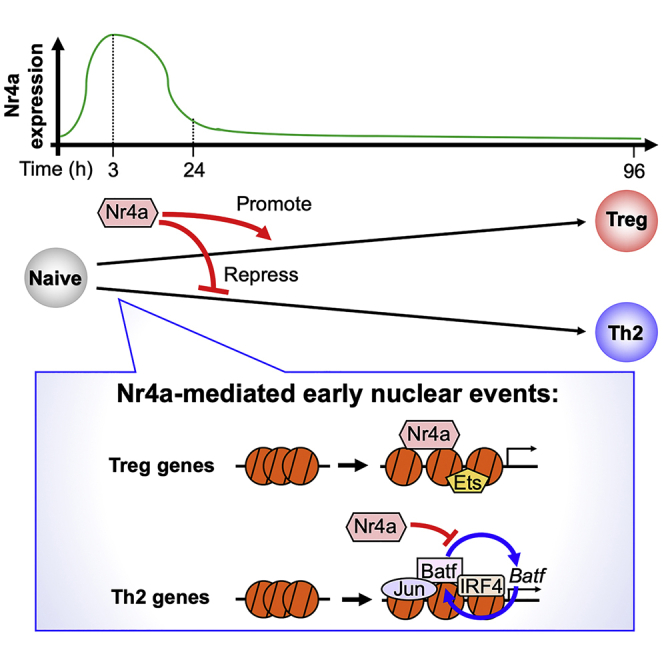

## Introduction

The CD4^+^ T cell lineage, comprising immune-activating helper T (Th) cell subsets, including Th1, Th2, Th17, and Tfh, and immune-suppressing regulatory T (Treg) cells, plays pivotal roles in mounting immune responses against diverse types of antigens, while preventing adverse immune responses against self- and commensal-antigens (Zhu et al., 2010). Dysregulated Th/Treg differentiation leads to various immune-associated diseases, including autoimmune diseases, allergies, and cancer.

Th1, Th2, Th17, Tfh, and Treg cell subsets are characterized by their unique expression of transcription factor sets, including T-bet, Gata3, Rorγt, Bcl-6, and Foxp3, respectively. Th and Treg cell differentiation is initiated from naive CD4^+^ T (naive T) cells, immediately after their recognition of antigen and cytokine stimulation, and their differentiation proceeds progressively. Differentiation of each Th and Treg subset culminates in the stabilized expression of their lineage-specifying transcription factors listed above (Kanno et al., 2012). The molecular events that govern the differentiation of Th and Treg cell subsets have been extensively analyzed. Fate decisions for the different Th and Treg subsets are largely dependent on the cytokine milieu surrounding the naive T cells (Zhu et al., 2010). For example, in Treg cell differentiation, TGF-β and IL-2 stimulation are indispensable as they activate the transcription factors Smads and Stat5, respectively (Chen et al., 2003; Takimoto et al., 2010; Zorn et al., 2006). Smads and Stat5 positively regulate the expression of *Foxp3* by acting on promoter or intronic enhancers present in the *Foxp3* locus (Burchill et al., 2007; Feng et al., 2014; Tone et al., 2008). In contrast to cytokine signaling, T cell receptor (TCR) signaling is commonly required for the differentiation of Th and Treg subsets; thus, TCR signaling is less well characterized as a skewing factor. Furthermore, in contrast to the crucial role of TCR signaling in Treg cell development in the thymus, its role in the specification of peripheral Treg (pTreg) cells is still unclear. However, accumulating evidence has shown that the strength and duration of TCR signaling affect the fate of naive T cells (Nakayama and Yamashita, 2010). It has been reported that weak avidity of TCR stimulation favors Th2 cell differentiation (Constant et al., 1995; Hosken et al., 1995).

Among the TCR signal-responsive transcription factors, the Nr4a family of nuclear orphan receptors has been shown to be highly dependent upon TCR stimulation (Ashouri and Weiss, 2017; Cheng et al., 1997; Moran et al., 2011; Sekiya et al., 2011). All members of the Nr4a family, viz., Nr4a1, Nr4a2, and Nr4a3, are rapidly induced by TCR stimulation. In previous reports, we have shown that Nr4a factors are able to induce Foxp3, thereby playing a crucial role in Treg cell development in the thymus (Sekiya et al., 2011, 2013, 2018). Further, mice that lacked all Nr4a family members died soon after birth due to severe autoimmunity. We also revealed that Nr4a factors are expressed at high levels in mature Treg cells, thereby maintaining the lineage stability of Treg cells (Sekiya et al., 2015). In addition, we and other groups have recently reported that Nr4a factors promote the exhaustion of T cells (Chen et al., 2019; Liu et al., 2019). Regarding the roles of Nr4a factors in peripheral CD4^+^ T cell differentiation, it was reported that Nr4a2-deficient naive T cells show attenuated Treg cell differentiation (Sekiya et al., 2011) and that Nr4a1-deficient naive T cells show accelerated production of IFN-γ upon activation (Liu et al., 2019). However, a comprehensive understanding of the roles of Nr4a factors in the peripheral differentiation of Th and Treg subsets from naive T cells is still lacking.

In this study, we investigated the roles of Nr4a factors in Th and Treg cell differentiation from naive T cells. Nr4a factors were found to promote Treg cell differentiation and repress Th1 and Th2 differentiation. During Treg cell differentiation, all Nr4a factors were transiently induced in naive T cells immediately after TCR stimulation, whereby they mediated epigenetic changes directly or by cooperating with other transcription factors. With the ability to promote Treg differentiation and repress Th2 differentiation, Nr4a factors suppressed allergic airway inflammation, mediating oral tolerance. We further showed that pharmacological activation of an engineered Nr4a molecule prevented the pathogenesis of allergic airway inflammation, thus, supporting the proposal that Nr4a factors can be therapeutic targets for inflammatory diseases.

## Results

### Nr4a factors regulate peripheral Th/Treg cell differentiation

In Nr4a1a2a3 triple knockout (Nr4a-TKO) mice, almost all CD4^+^ T cells show an activated phenotype, preventing the isolation of Nr4a-TKO naive T cells. Thus, to isolate Nr4a-TKO naive T cells, we constructed mixed bone marrow chimeras that were transferred Ly5.1^+^ wild-type (WT) and Ly5.2^+^ Nr4a-TKO cells (Figure S1A). In this environment, Treg cells develop from the WT counterpart and repress the activation of Nr4a-TKO cells, enabling the isolation of Nr4a-TKO naive T cells. WT naive T cells were also isolated from bone marrow chimeras (Figure S1B).

**Figure 1:**
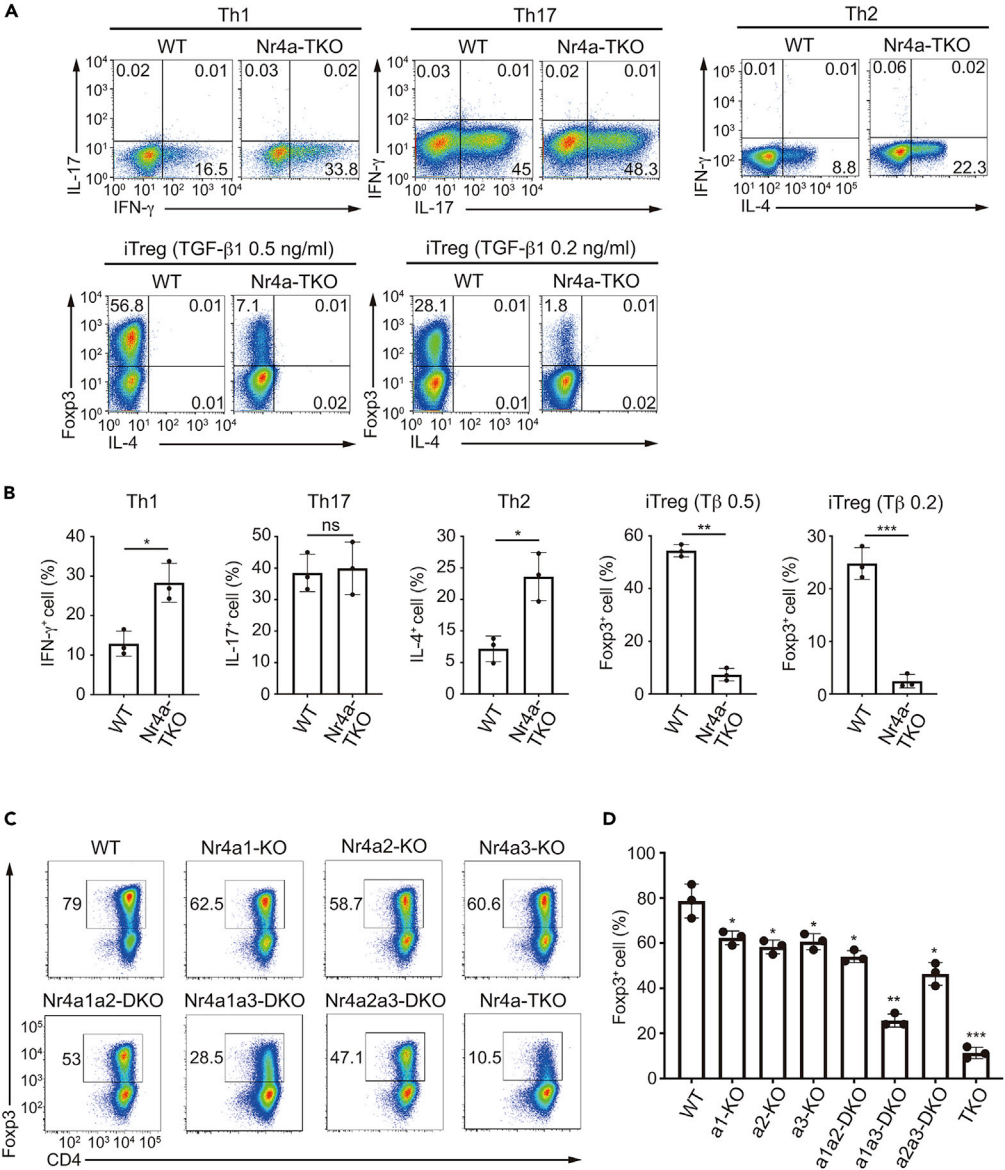
Nr4a factors are important for the induction of iTreg cells and the repression of Th1 and Th2 cell differentiation from naive T cells *in vitro* (A) Flow cytometry profiles of wild-type (WT) and Nr4a triple knockout (TKO) naive T cells cultured under the indicated conditions. Cells cultured under Th1, Th2, and Th17 conditions were analyzed 5 hr after restimulation with PMA + ionomycin. (B) Quantification of the result in (A). (C) Flow cytometry profiles of WT and Nr4a-single knockout, double knockout, and TKO naive T cells cultured under induced Treg (iTreg) cell-skewing conditions (with 0.5 ng/mL TGF-β1). (D) Quantification of the result in (C). Black dots represent individual values. Vertical bars and the error bars indicate mean and SD, respectively (B and D). Data are representative of 3 independent experiments with n = 3 (A)-(D). ∗p < 0.05; ∗∗p < 0.01; ∗∗∗p < 0.005. Unpaired Student's *t*-test (B), one-way ANOVA with Bonferroni test (D). In (D), p values on each bar represent the ones in comparison to WT control. See also Figure S2.

First, we compared the differentiation of WT and Nr4a-TKO naive T cells under conditions skewing to each Th and induced Treg (iTreg) cell subset *in vitro*. As shown in Figures 1A and 1B, Nr4a-TKO naive T cells showed accelerated differentiation to Th1 and Th2 cells, while showing attenuated differentiation to iTreg cells, particularly at lower concentrations of TGF-β1. Th17 differentiation was not affected in Nr4a-TKO cells. Further, the Nr4a-TKO cells were not skewed to Th1 or Th2 cells at the naive T cell stage, as we did not observe IFN-γ and IL-4 expression under Th0 conditions (Figure S2A). We also evaluated the differentiation of Nr4a-TKO naive T cells on a fixed TCR repertoire (the OT-II transgenic TCR) obtained by crossing Nr4a-TKO mice with Rag2^-/-^ OT-II TCR-Tg mice (hereafter referred to as “Nr4a-TKO-OT-II-Rag2^-/-^ mice”) (Figure S2B). Since a cognate antigen for OT-II TCRs, i.e., ovalbumin (OVA), is not present in mice, all CD4^+^ T cells are maintained in a naïve state even in Nr4a-TKO-OT-II-Rag2^-/-^ mice. By comparing the differentiation of WT and Nr4a-TKO OT-II naive T cells *in vitro*, we observed accelerated differentiation of Th1 and Th2 cells again, along with an attenuated iTreg cell differentiation of Nr4a-TKO cells (Figure S2B).

Subsequently, we analyzed the functional redundancy among the Nr4a family members in iTreg cell differentiation (Figures 1C and 1D). The single knockout, Nr4a1a2 double knockout (DKO), and Nr4a2a3-DKO naive T cells showed only slightly attenuated differentiation to iTreg cells. In contrast, the Nr4a1a3-DKO naive T cells showed attenuated iTreg cell differentiation, and Nr4a-TKO naive T cells exhibited further attenuated differentiation. These results show that Nr4a family members redundantly play important roles in iTreg cell differentiation. For all subsequent experiments, we compared Nr4a-TKO cells with WT cells, without analyzing cells individually deficient for Nr4a1, Nr4a2, and Nr4a3, and their DKOs.

**Figure 2:**
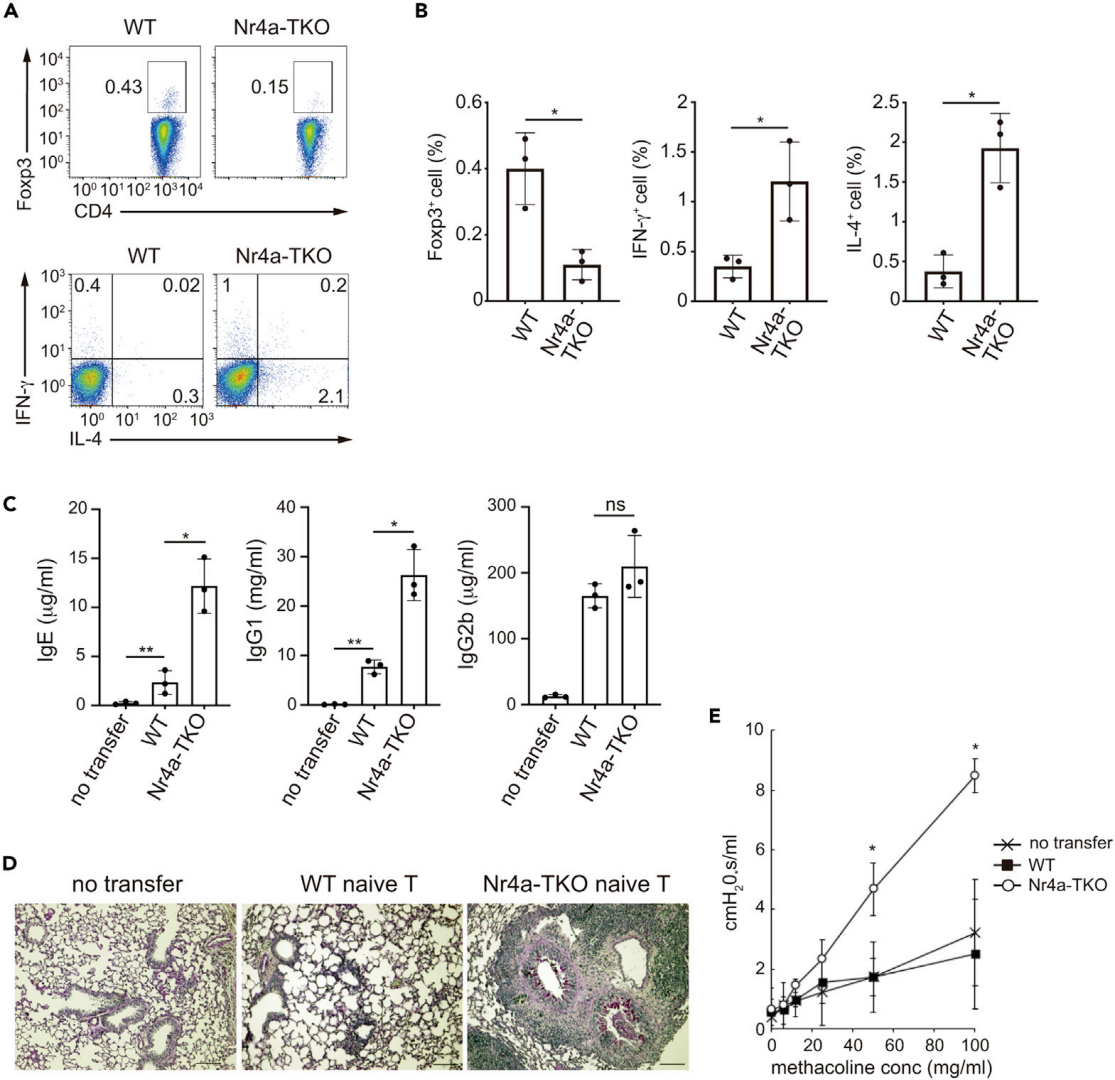
Nr4a factors are important for the induction of pTreg cells and the repression of Th1 and Th2 cell differentiation from naive T cells *in vivo* (A) Flow cytometry profiles of total CD3^+^CD4^+^ cells from spleens of TCRβ^−/-^ recipient mice that received WT and Nr4a-TKO naive T cells. (B) Quantification of the result in (A). Black dots represent individual values. Vertical bars and the error bars indicate mean and SD, respectively. (C) Titers of IgE, IgG1, and IgG2b in sera collected from untreated TCRβ^−/-^ mice and TCRβ^−/-^ mice receiving WT and Nr4a-TKO naive T cells (n = 3 per group). Black dots represent individual values. Vertical bars and the error bars indicate mean and SD, respectively. (D) PAS staining of lung sections from untreated TCRβ^−/-^ mice and TCRβ^−/-^ mice receiving WT and Nr4a-TKO naive T cells. Scale bars, 100 μm. (E) flexiVent system-mediated airway resistance measurement in response to methacholine challenge in untreated TCRβ^−/-^ mice and TCRβ^−/-^ mice receiving wild-type and Nr4a-TKO naive T cells. Each marker and the error bar denote mean and SD, respectively. Data represent three independent experiments, n = 3 per group (A)-(E). ∗p < 0.05; ∗∗p < 0.01. Unpaired Student's *t*-test (B), one-way ANOVA with Bonferroni test (C), two-way ANOVA with Sidak test (E). See also Figure S2.

Next, we investigated the differentiation of Nr4a-TKO naive T cells *in vivo*. We adoptively transferred polyclonal Nr4a-TKO naive T cells into TCRβ^-/-^ mice, which lack αβT cells, but have B cells. Analysis of CD4^+^ T cells isolated from the spleen of the recipient mice 30 days after transfer revealed that Nr4a-TKO naive T cells showed attenuated differentiation to pTreg cells but showed augmented differentiation to Th1 and Th2 cells (Figures 2A and 2B). Serum IgE and IgG1 levels were elevated in mice that received Nr4a-TKO cells, indicating augmented Th2-type immune reactions (Figure 2C). Histopathological examination of lung sections showed airway inflammation and mucus secretion from the airway epithelium (Figure 2D). Recipients of Nr4a-TKO naive T cells also showed accelerated airway hyperresponsiveness to methacholine, indicating the development of allergic asthma symptoms (Figure 2E). It is known that the adoptive transfer of naive T cells into lymphopenic mice, such as the TCRβ^-/-^ mice used in this study, elicits chronic colitis, which is associated with Th1 cell activity (Powrie et al., 1994). Indeed, both WT and Nr4a-TKO naive T cell-recipient TCRβ^-/-^ mice developed colitis (Figure S2D). However, we did not observe exacerbated colitis in the recipients of Nr4a-TKO cells, compared with those of WT cells. Accordingly, the serum level of IgG2b, a Th1-driven Ig isotype, was equivalent between recipients of WT and Nr4a-TKO cells (Figure 2C). Collectively, we observed that Nr4a-TKO naive T cells elicited allergic asthma symptoms with elevated Th2-type immune reactions, in addition to the Th1-driven pathology that are usually observed in the lymphopenic mice that received WT naive T cells. Although it was unexpected that Th1-driven pathology was not exacerbated in recipients of Nr4a-TKO cells despite their enhanced Th1 cell differentiation both *in vitro* and *in vivo*, related phenomenon was observed in other mice models of infection and autoimmune diseases, in which both Th1 and Th2 responses co-exist, whereas Th2 responses dominate functionally (Lenschow et al., 1996; Scott et al., 1994). Th2 responses elicited by Nr4a-TKO naive T cells possibly influenced the effector phase of the Th1 response (Wurtz et al., 2004).

We further examined the *in vivo* differentiation of Nr4a-TKO naive T cells, on a fixed TCR repertoire (OT-II transgenic TCR). We co-transferred congenic WT (Ly5.1) and Nr4a-TKO (Ly5.2) naive T cells at a 1:1 ratio into Rag2^-/-^ recipients and stimulated the mice under tolerizing conditions by oral administration of OVA (Figure S2C). This protocol is known to induce pTreg cells in the intestine (Mucida et al., 2005). Neither pTreg cells, nor Th1 or Th2 cells differentiated without OVA administration (Figure S2C). Conversely, pTreg cells differentiated from WT cells upon oral administration of OVA (Figure S2C). However, pTreg cell induction was substantially attenuated in Nr4a-TKO cells, while exhibiting accelerated differentiation to Th1 and Th2 cells.

### Nr4a factors transcriptionally regulate iTreg cell differentiation at an early phase

**Figure 3:**
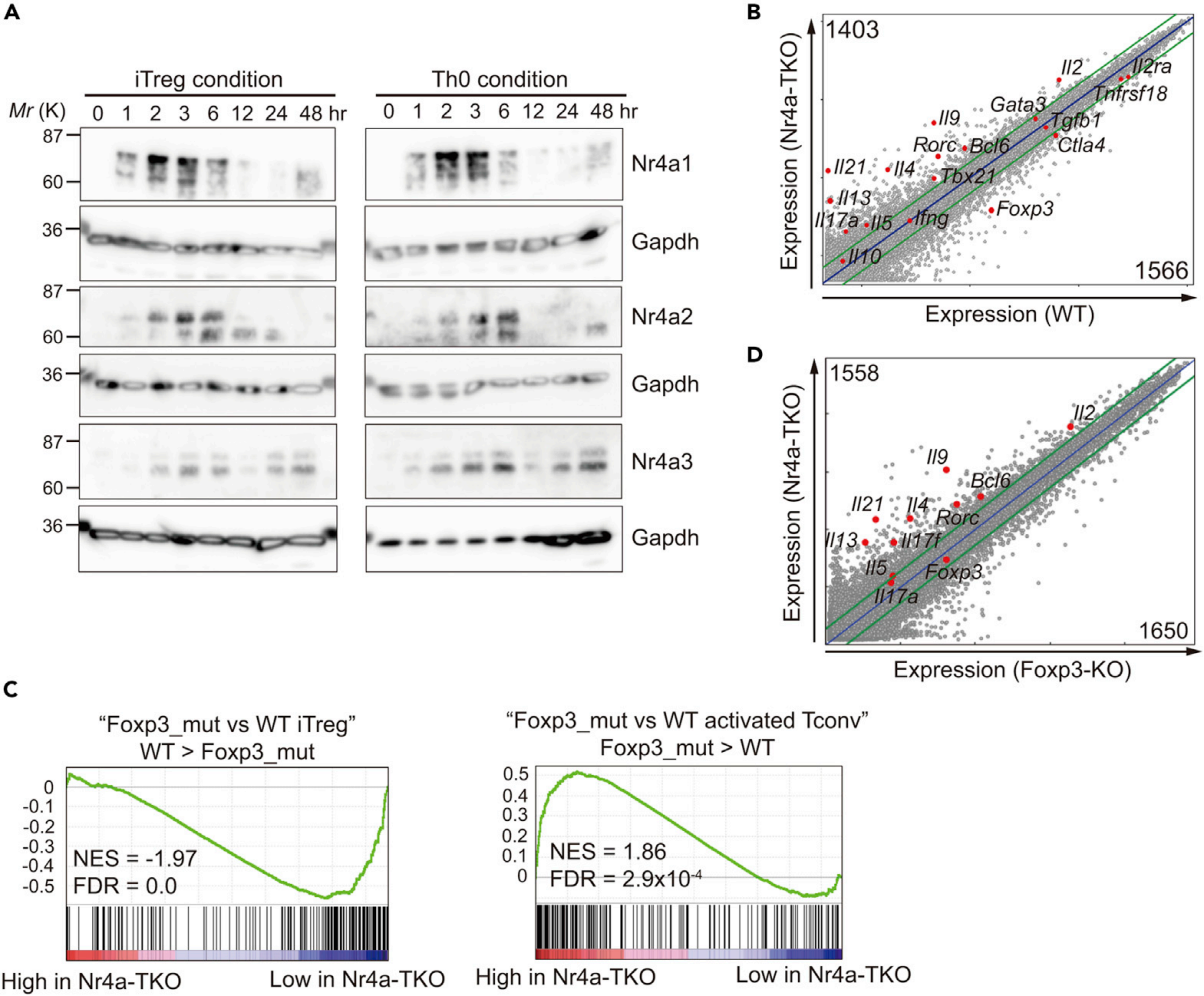
Transcriptional regulation of iTreg cells by Nr4a factors (A) Western blot analysis showing the time course of Nr4a family protein expression under indicated culture conditions. (B) mRNA expression profiles of wild-type (WT, horizontal axis) and Nr4a triple-knockout (TKO, vertical axis) induced Treg (iTreg) cells at 24 hr, analyzed by microarray. Selected Th- and Treg-regulatory genes are highlighted as red dots. A number of genes differentially expressed (|log_2_| > 1) are shown. (C) GSEA enrichment plot for the indicated MSigDB Hallmarks against the microarray data of WT iTreg at 24 hr vs. Nr4a-TKO iTreg at 24 hr. NES, normalized enrichment score; FDR, false discovery rate. (D) Expression profiles of Foxp3-knockout (KO, horizontal axis) and Nr4a-TKO (vertical axis) iTreg cells at 24 hr, analyzed by microarray. Selected Th- and Treg-regulatory genes are highlighted as red dots. A number of genes differentially expressed (|log_2_| > 1) are shown.

Next, we examined how the Nr4a factors regulate the differentiation of CD4^+^ T cells. Nr4a family members are known as the “immediate early genes” whose expression is induced immediately after TCR stimulation (Cheng et al., 1997). In addition, expression of Nr4a factors in T cells is specifically regulated by TCR signaling, showing little, if any, response to cytokine signaling and Th/Treg skewing conditions (Ashouri and Weiss, 2017; Moran et al., 2011; Sekiya et al., 2011). Western blotting of lysates of CD4^+^ T cells cultured under Th0 or iTreg conditions revealed an equivalent expression pattern for all Nr4a factors between the two conditions (Figure 3A). Protein expression was induced immediately after stimulation, and the induction was transient, peaking at approximately 2-6 h after stimulation. Nr4a3 expression increased again after 24 h. Collectively, our findings suggest that Nr4a factors regulate CD4^+^ T cell differentiation at an early phase. Next, we compared gene expression between WT and Nr4a-TKO iTreg cells at 24 h by microarray analysis. As shown in Figure 3B, 1403 and 1566 genes were upregulated and downregulated, respectively, by more than two-fold in Nr4a-TKO cells. As expected, *Foxp3* was found among the downregulated genes in Nr4a-TKO cells (Figure 3B). In contrast, several cytokine genes associated with allergic inflammation, including *Il4*, *Il5*, *Il9*, *Il13*, and *Il21*, were found to be upregulated in the Nr4a-TKO cells. Gene set enrichment analysis (GSEA) revealed that the difference between WT and Nr4a-TKO iTreg cells was highly correlated with the difference between WT and Foxp3-KO iTreg cells (Figure 3C). This result suggests that one of the most important roles of Nr4a factors in iTreg cell differentiation is to positively regulate Foxp3 expression. Since Foxp3 is known to regulate a large proportion of the Treg cell transcriptional program (Gavin et al., 2007; Lin et al., 2007), we next attempted to distinguish Foxp3-dependent and -independent Nr4a factor-mediated gene regulatory events by comparing gene expression between Nr4a-TKO and Foxp3-KO iTreg cells. Foxp3-KO naive T cells were obtained from mixed bone marrow chimeras that were transferred Ly5.1^+^ WT and Ly5.2^+^ Foxp3-KO cells (Figure S1C). As shown in Figure 3D, expression of allergy-associated cytokine genes, including *Il4*, *Il5*, *Il9*, *Il13*, and *Il21*, was higher in Nr4a-TKO cells, even when compared with Foxp3-KO cells. Collectively, we showed that Nr4a factors transcriptionally regulate iTreg cell differentiation in part, by positively regulating Foxp3 expression, while repressing allergy-associated cytokine genes in a Foxp3-independent manner.

### Nr4a factors activate target chromatin sites directly or by promoting the activity of Ets factors

**Figure 4:**
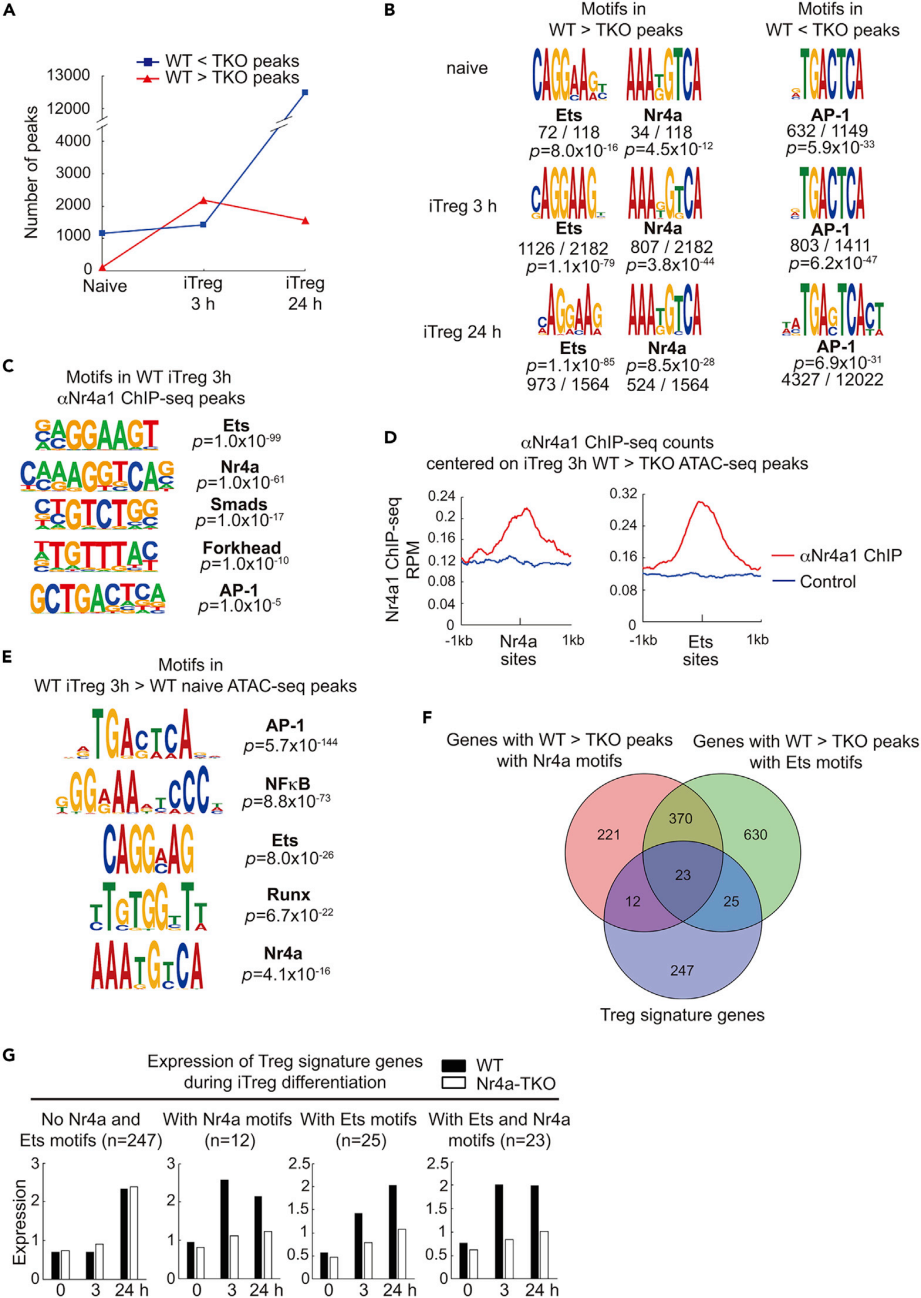
Chromatin regulation of iTreg cell differentiation by Nr4a factors (A) Number of differentially accessible ATAC-seq peaks between wild-type (WT) naive T and Nr4a triple-knockout (TKO) naive T, between WT iTreg at 3 hr and Nr4a-TKO iTreg at 3 hr, and between WT iTreg at 24 hr and Nr4a-TKO iTreg at 24 hr. (B) Motifs enriched in differentially accessible ATAC-seq peaks between WT naive T and Nr4a-TKO naive T, between WT iTreg at 3 hr and Nr4a-TKO iTreg at 3 hr, and between WT iTreg at 24 hr and Nr4a-TKO iTreg at 24 hr. Total number of differentially accessible peaks in each comparison and the number of peaks that contain the indicated motifs are shown with p values. (C) Motifs enriched in chromatin samples immunoprecipitated with anti-Nr4a1. (D) Normalized counts of anti-Nr4a1 and control (pre-immune sera) ChIP-seq reads, centered on Nr4a and Ets binding motifs in ATAC-seq peaks that were more accessible in WT iTreg at 3 hr than in Nr4a-TKO iTreg at 3 hr. (E) Motifs enriched in differentially accessible ATAC-seq peaks between WT naive T and WT iTreg at 3 hr. (F) Venn diagram of Treg signature genes and genes annotated from Nr4a or Ets motif-containing ATAC-seq peaks that were more accessible in WT iTreg at 3 hr than in Nr4a-TKO iTreg at 3 hr. (G) Microarray analysis of mRNA expression of Treg signature genes that were classified as shown in (F). Expression levels in WT (black bars) and Nr4a-TKO (white bars) naive T, iTreg at 3 hr, and iTreg at 24 hr. See also Figures S3 and S4.

We investigated how Nr4a factors epigenetically regulate CD4^+^ T cell differentiation. We first performed an assay for transposase-accessible chromatin (ATAC)-seq, on WT and Nr4a-TKO naive T and iTreg cells. iTreg cells were examined at 3 h, the time when the protein expression of Nr4a factors peaks, and at 24 h, the time when the expression of Nr4a factors ceases. As shown in Figure 4A, during differentiation from naive T to iTreg cells at 3 h, the number of genomic regions more accessible in Nr4a-TKO cells than in WT cells (termed “Nr4a-TKO-specific peaks”) increased slightly. In contrast, the number of genomic regions more accessible in WT cells than in Nr4a-TKO cells (termed “WT-specific peaks”) increased substantially (Figure 4A). We found enrichment of binding motifs for Nr4a factors and Ets family transcription factors among the WT-specific peaks in iTreg cells at 3 h (Figure 4B). Collectively, considering that expression of Nr4a factors peaks around 3 h, it was suggested that one of the roles of Nr4a factors in Treg cell differentiation is to activate target chromatin sites, either directly or by supporting the function of Ets factors.

To further investigate the molecular mechanisms by which Nr4a factors activate target chromatin sites in the early phase of iTreg cell differentiation, we performed ChIP-seq assays on WT iTreg cells at 3 h, utilizing antisera developed against Nr4a1. In agreement with the results of the ATAC-seq, the Ets and Nr4a binding motifs were the first and second most significantly enriched sequences, respectively (Figure 4C). In addition, we also detected motifs for Smads and Forkhead factors, implying possible interactions between these transcription factors and Nr4a. Integrative analysis of ChIP-seq and ATAC-seq revealed an enrichment of Nr4a1 ChIP-seq reads around the Nr4a binding motifs found in the WT-specific ATAC-seq peaks, as expected (Figure 4D, left). Importantly, we also detected an enrichment of anti-Nr4a1 ChIP-seq reads around the Ets binding motifs found in the WT-specific ATAC-seq peaks (Figure 4D, right). The latter result shows that Nr4a factors are recruited to the target sites of Ets factors, thereby supporting chromatin activation. Thus, we next investigated whether Nr4a factors physically interact with the Ets factors. Among the various Ets family members, we analyzed Ets-1, Ets-2, Fli-1, and Pu.1, as these factors have been shown to bind the AGGAAG sequence found in the WT-specific ATAC-seq peaks and are also known to be expressed in CD4^+^ T cells. In this experiment, Flag-tagged Nr4a factors and T7-tagged Ets factors were ectopically co-expressed in 293T cells, and the lysates were immunoprecipitated with anti-T7 antibodies. A known interaction between Nr4a2 and Runx1 was examined as an experimental control (Sekiya et al., 2011). As shown in Figure S3, we observed the binding of Nr4a2 to Fli-1 and Pu.1, and detected a weak interaction between Nr4a3 and Fli-1. However, none of the examined Ets factors show an interaction with Nr4a1.

The Nr4a binding motif was among the five motifs significantly enriched in chromatin regions that are more accessible in WT iTreg cells at 3 h than in WT naive T, confirming the importance of Nr4a factors in the early phase of iTreg cell differentiation (Figure 4E). Next, we classified the “Treg signature genes” (Hill et al., 2007), a set of genes specifically expressed in Treg cells, according to the presence of WT-specific ATAC-seq peaks with Nr4a or Ets binding motifs. Among the 307 Treg signature genes, 12, 25, and 23 genes were found to have WT-specific ATAC-seq peaks with Nr4a motifs, Ets motifs, and both, respectively (Figures 4F and S4). We then analyzed the expression of genes in each group during iTreg cell differentiation by microarray analysis. We found that the expression of genes without such motifs did not increase at 3h but increased at 24 h during iTreg cell differentiation (Figure 4G, “No Nr4a and Ets motifs”). These genes were induced in Nr4a-TKO cells as well (Figure 4G, white bars). In contrast, genes with Nr4a or Ets motif-containing WT-specific ATAC-seq peaks were induced even at 3h. Genes in these groups were not induced in Nr4a-TKO cells, indicating their dependence on Nr4a factors. Collectively, these results show that Nr4a factors induce early responsive genes in the Treg cell-specific gene set, either directly or by supporting the function of Ets factors.

### Nr4a factors interrupt a positive feedback loop for Batf3 expression to repress Th2 genes

**Figure 5:**
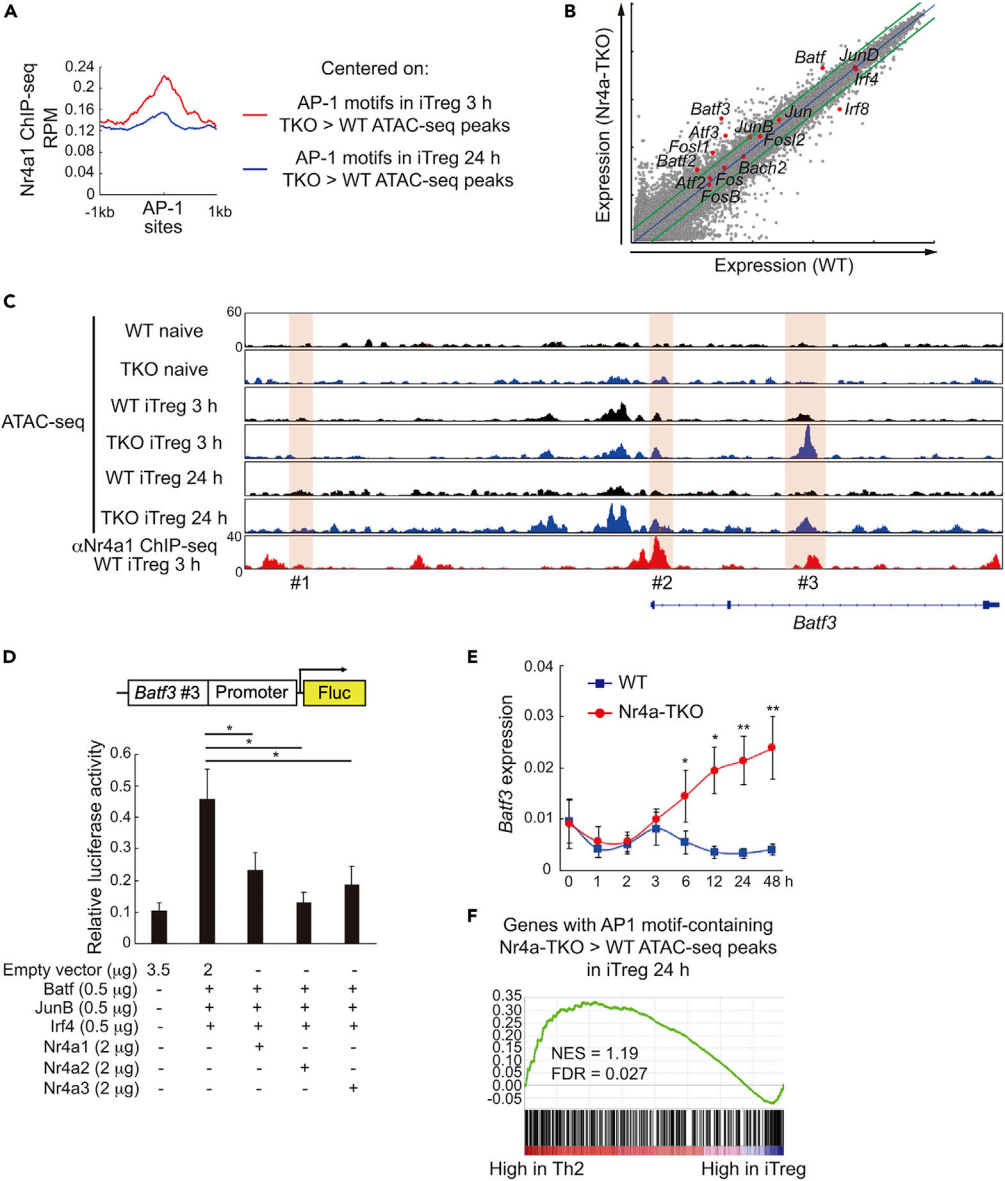
Nr4a factors interrupt positive feedback amplification of *Batf3* (A) Normalized anti-Nr4a1 ChIP-seq reads, centered on AP-1 binding motifs in ATAC-seq peaks that were more accessible in Nr4a triple knockout (TKO) iTreg cells than in WT induced Treg (iTreg) cells at 3 hr (red) and 24 hr (blue). (B) mRNA expression profiles of WT (horizontal axis) and Nr4a-TKO (vertical axis) iTreg at 24 hr, analyzed by microarray. Selected AP-1-associated genes are highlighted as red dots. (C) Genome browser view of ATAC-seq and anti-Nr4a1 ChIP-seq profile maps at *Batf3* locus. Highlights indicate the three target sites that were suggested to mediate the positive feedback amplification of *Batf3* by Batf factors. Vertical scales are noted. (D) Top: A schematic of luciferase reporter construct (Batf3-Luc), which incorporates the *Batf3* #3 region and the *Batf3* promoter. Bottom: Luciferase reporter activities of Batf3-Luc in Jurkat cells transfected with plasmids encoding AP-1 factors (Batf, JunB, Irf4, 0.5 μg each) and Nr4a factors (Nr4a1, Nr4a2, and Nr4a3, 2 μg each). Total amounts of plasmids were adjusted with an empty plasmid. Relative luciferase values normalized to Renilla luciferase activity from co-transfected pRL-tk are shown. (E) Time course of mRNA levels of *Batf3* in wild-type and Nr4a-TKO naive T cells cultured under iTreg conditions. Results are presented relative to expression of the control gene *Hprt*. (F) GSEA of *in vitro* differentiated Th2 cells and iTreg cells (Wei et al., 2009). Gene set used: genes with AP-1 motif-containing ATAC-seq peaks that were more accessible in Nr4a-TKO iTreg at 24 hr than in the WT iTreg at 24 hr. Data represent mean ± SD of two (E) and three (D) independent experiments, performed in triplicate. ∗p < 0.05; ∗∗p < 0.01 (One-way ANOVA with Bonferroni test (D), two-way ANOVA with Sidak test (E)).

In contrast to the changes that occurred during the first 3 h of iTreg cell differentiation, analysis of changes that occurred between 3 to 24 h of differentiation revealed that the number of Nr4a-TKO-specific ATAC-seq peaks substantially increased, whereas the number of WT-specific peaks decreased slightly (Figure 4A). Furthermore, we found an enrichment of binding motifs for AP-1 family transcription factors among these Nr4a-TKO-specific peaks (Figure 4B). Considering the decrease in expression of Nr4a factors from 3 to 24 h during iTreg cell differentiation, it may be inferred that Nr4a factors play a role at around 3 h, which represses the later increase in AP-1 activity. Thus, we next investigated the Nr4a factor-mediated gene repression events during iTreg cell differentiation, by focusing on the regulation of AP-1 factors. Although the AP-1 motif was found to be enriched in the Nr4a1 ChIP-seq peaks, its significance was far less than that of the Ets and Nr4a factors (Figure 4C). Subsequently, we analyzed the Nr4a1 ChIP-seq reads around the AP-1 motifs in the Nr4a-TKO-specific ATAC-seq peaks of iTreg cells at 3 h and 24 h (Figure 5A). It was found that AP-1 motifs in Nr4a-TKO-specific ATAC-seq peaks of iTreg cells at 3h were more extensively bound by Nr4a1 than those of iTreg cells at 24 h. This result suggests that Nr4a factors directly repress some key AP-1-mediated events at approximately 3 h, which prevents the increase in AP-1 activity later. Comparison of the mRNA expression of AP-1-associated molecules between WT and Nr4a-TKO iTreg cells at 24 h revealed remarkable upregulation of all Batf family members, viz., *Batf*, *Batf2*, and *Batf3*, in Nr4a-TKO cells (Figure 5B). This result led us to focus on a previous study that implicated a positive feedback loop for Batf factor expression, showing that Batf factors bind to regulatory regions of *Batf* and *Batf3* genes, and positively regulate their own expression (Kuwahara et al., 2016). We analyzed chromatin accessibility and Nr4a1 binding at the *Batf3* locus (Figure 5C). Among the three regions that are bound by Batf factors, we found accelerated accessibility at the #3 locus in Nr4a-TKO iTreg cells. This region also showed an enrichment of anti-Nr4a1 ChIP fragments. We then analyzed the effects of Nr4a factors on Batf3 expression by luciferase reporter assay, utilizing a reporter construct containing the *Batf3* promoter and the #3 locus (Figure 5D). Transduction of this reporter construct into Jurkat T cells, along with expression plasmids for Batf, JunB, Irf4, and Nr4a factors, revealed that all Nr4a factors suppressed the Batf/JunB/Irf4-mediated activation of reporter expression. We next compared *Batf3* expression between WT and Nr4a-TKO cells during iTreg differentiation. As shown in Figure 5E, *Batf3* expression was immediately downregulated upon activation, but recovered at 3 h, in both WT and Nr4a-TKO cells. However, although the expression was downregulated in WT cells thereafter, it was gradually upregulated in the Nr4a-TKO cells (Figure 5E). Summarizing the above results, we propose the following mechanism: Nr4a factors interrupt a positive feedback loop for Batf factor expression at an early stage of iTreg cell differentiation. Finally, we analyzed the effect of the aberrant increase in AP-1 activity on Nr4a-TKO iTreg cell differentiation using GSEA. First, we generated a set of genes that showed Nr4a-TKO-specific ATAC-seq peaks with AP-1 motifs. Analyzing the correlations of this gene set with genes that are differentially expressed between Th2 and iTreg cells (Wei et al., 2009), we found that the gene set was significantly enriched among genes that were highly expressed in Th2 cells (Figure 5F). This result shows that aberrant AP-1 activity in Nr4a-TKO iTreg cells drove a Th2 cell transcriptional program.

### Nr4a factors suppress airway inflammation and mediate oral tolerance

**Figure 6:**
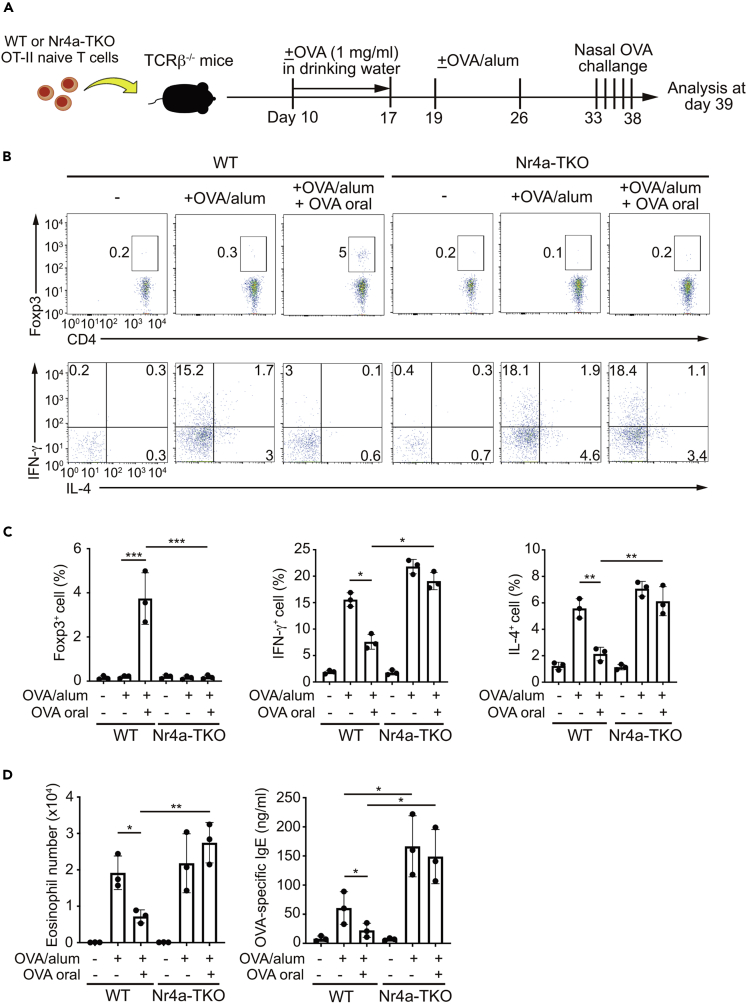
Roles of Nr4a factors in allergic airway inflammation pathogenesis and induction of oral tolerance (A) A schematic of the experiment performed. (B) Flow cytometry profiles of total CD3^+^CD4^+^ cells from spleens of TCRβ^−/-^ mice that received wild-type (WT) or Nr4a triple-knockout (TKO) OT-II naive T cells, as indicated. (C) Quantification of the results in (B). (D) Eosinophil number and titers of OVA-specific IgE in BAL of TCRβ^−/-^ mice that received WT or Nr4a-TKO OT-II naive T cells, as indicated. Data are representative of two independent experiments, n = 3 each (B, C, and D). Black dots represent individual values. Vertical bars and the error bars indicate mean and SD, respectively (C and D). ∗p < 0.05; ∗∗p < 0.01; ∗∗∗p < 0.005, one-way ANOVA with Bonferroni test (C and D).

The studies described above revealed that Nr4a factors play important roles in inducing Treg cells while repressing Th2 cell differentiation. Thus, we subsequently assessed the impact of Nr4a factors on allergic airway inflammation pathogenesis. In this study, we also investigated the role of Nr4a factors in the induction of oral tolerance. This experiment was modified from the one performed by Curotto de Lafaille et al. (Curotto de Lafaille et al., 2008). In this study, mice were transferred with WT or Nr4a-TKO OT-II naive T cells, and then immunized twice with OVA combined with alum as an adjuvant (Figure 6A). One week after the second OVA/alum immunization, mice were nasally challenged with OVA. Some mice received OVA orally for 1 week, until 2 days before the first OVA/alum immunization. As shown in Figures 6B and 6C, both WT and Nr4a-TKO cells differentiated into Th1 and Th2 cells following OVA/alum immunization, with slightly accelerated differentiation from Nr4a-TKO cells. Recipients of WT and Nr4a-TKO cells both exhibited elevated bronchoalveolar lavage (BAL) eosinophil number and OVA-specific IgE levels by OVA/alum immunization, whereas recipients of Nr4a-TKO cells showed higher levels of OVA-specific IgE (Figure 6D). Importantly, WT cells, but not Nr4a-TKO cells, differentiated into pTreg cells upon oral OVA administration, with a concomitant reduction in Th1 and Th2 cell differentiation (Figures 6B and 6C). Furthermore, in recipients of WT cells, but not Nr4a-TKO cells, both BAL eosinophil number and OVA-specific IgE levels were reduced following oral OVA administration (Figure 6D). Collectively, Nr4a factors showed the ability to suppress allergic airway inflammation and to promote oral tolerance by stimulating pTreg cell differentiation.

### Pharmacological activation of an engineered Nr4a molecule prevented airway inflammation

**Figure 7:**
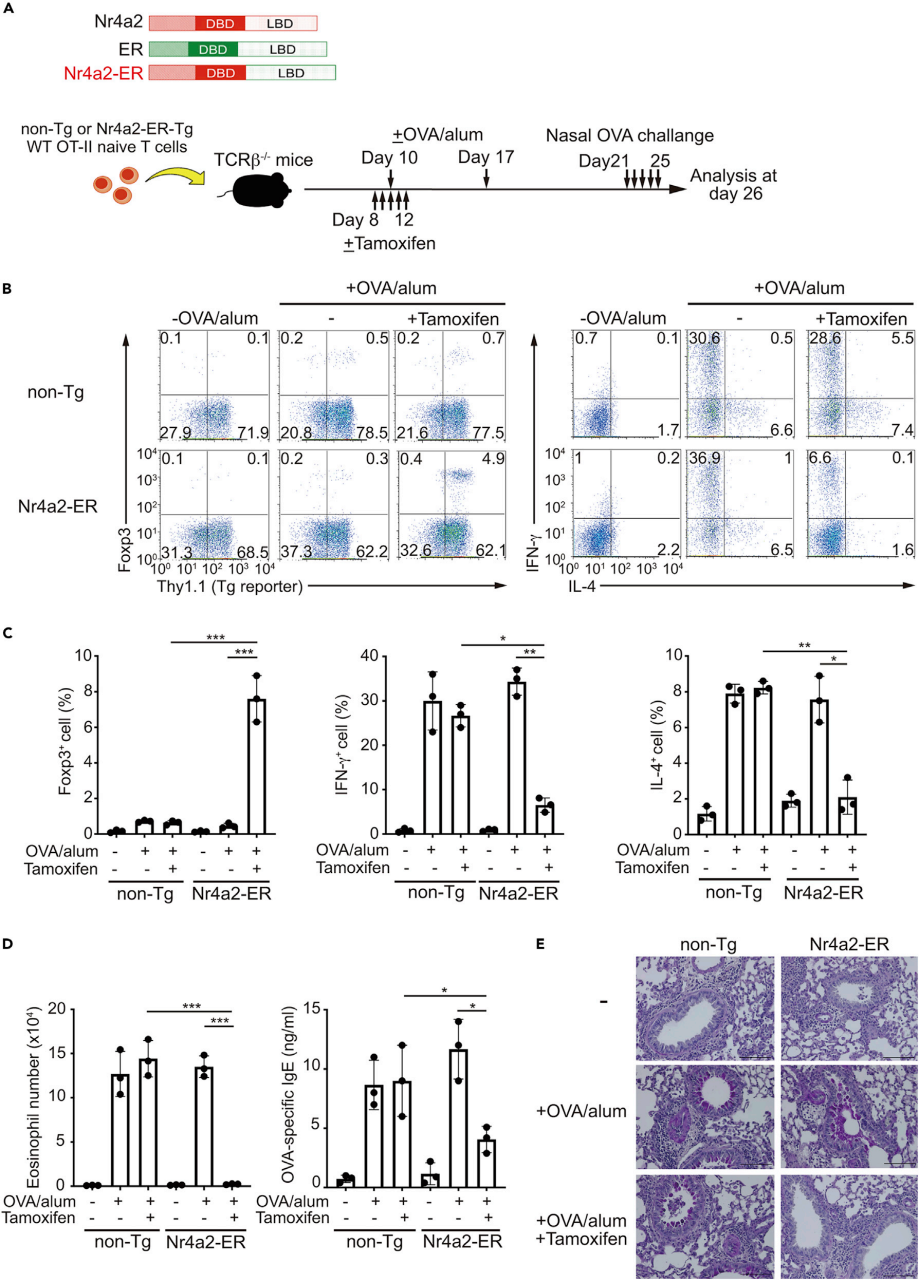
Prevention of allergic airway inflammation by a Nr4a-ER chimeric molecule (A) Top: Structures of Nr4a2, estrogen receptor (ER), and the Nr4a2-ER chimeric molecule. DBD: DNA binding domain; LBD: ligand binding domain. Bottom: A schematic of the experiments performed. (B) Flow cytometry profiles of total CD3^+^CD4^+^ cells from spleens of TCRβ^−/-^ mice that received non-Tg or Nr4a2-ER-Tg wild-type (WT) OT-II naive T cells, as indicated. (C) Quantification of the results in (B). (D) Eosinophil number and titers of OVA-specific IgE in bronchoalveolar lavage of TCRβ^−/-^ mice that received non-Tg or Nr4a2-ER-Tg WT OT-II naive T cells, as indicated. (E) PAS staining of lung sections from TCRβ^−/-^ mice that received non-Tg or Nr4a2-ER-Tg WT OT-II naive T cells, as indicated. Scale bars, 100 μm. Data are representative of two independent experiments, n = 3 each (C and D). Black dots represent individual values. Vertical bars and the error bars indicate mean and SD, respectively (C and D). ∗p < 0.05; ∗∗p < 0.01; ∗∗∗p < 0.005, one-way ANOVA with Bonferroni test (C and D).

Finally, we sought to determine the potential of Nr4a factors as novel therapeutic targets for allergic airway inflammation. As effective pan-Nr4a activating chemicals are not available, we employed a previously reported chimeric molecule, in which the ligand binding domain (LBD) of Nr4a2 is replaced by the LBD of estrogen receptor (ER) (Lyszkiewicz et al., 2019; Sekiya et al., 2013) (Figure 7A). This engineered molecule (Nr4a2-ER) can be activated by tamoxifen, and can induce Foxp3 in CD4^+^ T cells upon activation (Sekiya et al., 2013). Nr4a2-ER-expressing WT OT-II naive T cells (called “Nr4a2-ER-Tg OT-II naive T cells”) were isolated from bone marrow chimeric mice, whose hematopoietic cells were reconstituted with cells transduced with an Nr4a2-ER-expressing retrovirus. Then, Nr4a2-ER-Tg OT-II naive T cells were transferred into TCRβ^-/-^ recipients, and the mice were immunized with OVA/alum. Mice were then nasally challenged with OVA to elicit allergic airway inflammation (Figure 7A). Tamoxifen was administered at the time of immunization, as Nr4a factors regulate the differentiation of CD4^+^ T cells at an early phase, and as Nr4a factors induce exhaustion at a later phase of T cell activation, which may complicate the interpretation of the results. As shown in Figures 7B–7E, OVA/alum immunization induced Th1 and Th2 cell differentiation, and a concomitant increase in BAL eosinophil number, OVA-specific IgE, and airway mucus secretion. Upon treatment with tamoxifen, Nr4a2-ER-Tg cells, but not non-Tg cells, induced Foxp3 expression in naive T cells, while suppressing Th1 and Th2 cell differentiation. Furthermore, reductions in BAL eosinophil number, OVA-specific IgE production, and airway mucus secretion were observed specifically in recipients of Nr4a2-ER-Tg cells upon treatment with tamoxifen (Figures 7D and 7E). Collectively, these results show that prophylactic activation of Nr4a factors can suppress airway inflammation pathogenesis by inducing pTreg cell differentiation, while repressing Th1 and Th2 cell differentiation.

## Discussion

Compared with the fate decision of developing CD4^+^ T cells in the thymus, which is largely determined by the strength of TCR signaling, the fate decision of peripheral naive T cells is less dependent on TCR signaling. Instead, peripheral naive T cells mainly depend on cytokine signaling for their fate choice. Thus, peripheral Th and Treg cell differentiation has been studied with special emphasis on cytokine signaling. Regarding this issue, the expression of Nr4a factors has been known to be exclusively regulated by TCR signaling, with little, if any, contribution from cytokine signaling (Ashouri and Weiss, 2017; Moran et al., 2011; Sekiya et al., 2011). However, in this study, we revealed that Nr4a factors play important roles in peripheral CD4^+^ T cell differentiation from naive T cells by promoting Treg cell differentiation, and by repressing Th1 and Th2 cell differentiation.

As a molecular mechanism that mediates the repression of Th2-associated genes during iTreg cell differentiation, we found that Nr4a factors inhibit the increase in AP-1 factor activity by interfering with a positive feedback loop for Batf factor expression. Batf/Jun AP-1 complexes have been reported to positively regulate Tfh, Th9, Th17, and Th2 cell differentiation (Bao et al., 2016; Betz et al., 2010; Ise et al., 2011; Jabeen et al., 2013; Schraml et al., 2009). Other studies have also reported AP-1 repression by Nr4a factors. Nr4a factors counteract AP-1 activity during T cell exhaustion, resulting in the repression of effector T cell transcription (Chen et al., 2019; Liu et al., 2019). As a molecular mechanism that mediates counteraction of AP-1 activity by Nr4a factors, one study reported that Nr4a factors interrupt access of AP-1 factors to their target sites during T cell exhaustion (Liu et al., 2019). A similar mechanism may be exerted during iTreg cell differentiation. In addition, Batf factors have been shown to repress Foxp3 expression by mediating repressive chromatin modifications (Zhang et al., 2018). Thus, the upregulated Batf factors may also directly contribute to the attenuated iTreg differentiation of Nr4a-TKO cells.

Although Nr4a factors interrupt the amplification of Batf factor expression, and repress Th2 cell differentiation, Th17 cell differentiation is also heavily dependent on Batf factors. However, we did not observe augmented Th17 cell differentiation in the Nr4a-TKO naive T cells. The fact that both Batf factors and Th17-associated genes, including *Rorc* and *Il17a*, were upregulated in Nr4a-TKO iTreg cells at 24 h suggests that Nr4a factors repress Th17 cell emergence during iTreg cell differentiation (Figure 3B). Thus, it is suspected that Nr4a factors have a Th17 cell-specific function that is not exerted during iTreg cell differentiation. In this aspect, it was reported that Nr4a2 positively regulates IL-17 expression, independent of Rorγt expression, during Th17 cell differentiation (Doi et al., 2008; Raveney et al., 2013). siRNA-mediated knockdown of Nr4a2 does not reduce Rorγt expression, but it represses the factors that are important in the later phase of Th17 cell differentiation, including Il-21 and IL-23R, thereby inhibiting IL-17 expression under Th17-skewing conditions (Raveney et al., 2013). Therefore, it is possible that the negative effect of Batf factor repression on Th17 cell differentiation by Nr4a factors was counterbalanced by the positive effect of Nr4a2 on IL-17 expression. In addition, although Th1 cell differentiation was augmented in Nr4a-TKO cells, differentiation of this cell subset is not dependent on Batf factors (Schraml et al., 2009). Thus, it is suggested that the repression of Th1 cell differentiation by Nr4a factors is mediated differently as compared with the repression of Th2 cell differentiation. Although we have not provided insights into the mechanism that explains this phenomenon, it is possible that repression of Th1 cell differentiation is mediated by a similar mechanism in which Nr4a factors induce dysfunctional T cells, wherein IFN-γ expression is also repressed (Chen et al., 2019; Liu et al., 2019). In addition, it is also possible that Nr4a factors negatively regulate Th1cell differentiation by repressing T-bet expression because the expression of *Tbx21*, the gene encoding T-bet, was elevated in Nr4a-TKO iTreg cells (Figure 3B).

Among the “Treg signature genes”, the genes regulated by Nr4a factors were found to be induced in the early phase of differentiation. To our knowledge, no previous study has classified Treg signature genes according to their expression kinetics. Gene ontology analysis revealed that the Nr4a-regulated Treg signature genes matched the biological process of “negative regulation of signal transduction” most significantly. Thus, one of the mechanisms by which Nr4a factors promote Treg cell differentiation might be the repression of inhibitory cytokine signaling or excessive TCR signaling that potentially inhibits differentiation (Chang et al., 2011; Yamane et al., 2005). Nr4a factors support the activity of Ets factors to activate a subset of Treg signature genes. Importantly, impaired development of Treg cells, as well as elevated Th2 cell activity were also observed in Ets-1-deficient mice (Mouly et al., 2010). A previous study reported cooperation between Nr4a and Ets factors in gene activation in human leukemia cells and found that NR4A1 regulates transcription by binding to enhancers that are co-enriched for Nr4a- and Ets factor-binding motifs, thereby promoting the recruitment of ERG, FLI-1 Ets factors, and p300 histone acetyltransferase (Duren et al., 2016). In this regard, although we found that a sizable fraction of WT cell-specific ATAC-seq peaks contained both Nr4a and Ets motifs, a majority of the sites contained only the Ets motifs (Figure 4F). Since the ChIP-seq results showed the localization of Nr4a factors to Ets motifs in WT-specific ATAC-seq peaks, we suggest that Nr4a factors support the function of Ets factors in a DNA binding-independent manner. This assumption is further supported by the attenuation of the expression of genes with only Ets motifs in Nr4a-TKO iTreg cells (Figure 4G). In this aspect, by investigating the physical interaction between Nr4a and Ets factors, we observed the interaction of Nr4a2 with Pu.1, as well as Nr4a2 and Nr4a3 with Fli-1. However, none of the examined Ets factors interacted with all Nr4a members. Although we do not rule out the possibility that the observed interactions have a significant meaning, it is also possible that physiologically meaningful interactions between Nr4a and Ets factors are fundamentally weak or transient, and are, thus, undetectable by co-immunoprecipitation. Another possibility is that the binding of Nr4a factors to motifs other than the AAA(T/G)GTCA sequence may potentially lead to underestimation of the co-binding of Nr4a and Ets factors (Hu et al., 2009; Perlmann and Jansson, 1995).

It was unexpected that the ATAC-seq results showed some differences between WT and Nr4a-TKO cells even at the naive T cell stage, as none of the Nr4a family members were detected at this stage. However, recent studies suggest that naive T cells are not a homogenous population. Instead, heterogeneity in the naive T cell population is generated according to the strength of “tonic” TCR signaling, a low-level stimulation by self-peptide–MHC complexes, which does not elicit canonical activation pathways. In particular, naive T cells with high tonic signaling, which are marked by higher levels of Nr4a1 expression, confer higher propensities toward iTreg cell differentiation (Martin et al., 2013; Zinzow-Kramer et al., 2019). Thus, Nr4a factors, which are expressed at a level undetectable by even western blotting, may modulate epigenetic status in naive T cells, particularly in the cells that received high tonic signaling. The contribution of Nr4a factors to the altered phenotype of naive T cells will be revealed in future studies. However, even with such altered epigenetic status, Nr4a-TKO naive T cells were not skewed to particular Th cell subsets (Figure S2A).

Altogether, we revealed that Nr4a factors play important roles in the induction of Treg cells, as well as in the repression of Th1 and Th2 cell differentiation from naive T cells at an early phase of their differentiation, which are linked to TCR signaling. We also revealed the potential of Nr4a factors as molecular targets for the treatment of allergic diseases. Since plasticity among Th and Treg cell subsets is progressively lost during differentiation (Ansel et al., 2003; Bird et al., 1998; Murphy et al., 1996), early differentiation phases can be an ideal time point for therapeutic intervention. Mice that lacked all Nr4a family members in the T cells died early due to severe systemic autoimmunity (Sekiya et al., 2013). As one of the central players in the suppression of autoimmunity, Nr4a factors have been revealed to mediate various aspects of immune tolerance, including tTreg cell development, negative selection of self-reactive thymocytes, and the induction of exhaustion during chronic stimulation (Calnan et al., 1995; Chen et al., 2019; Liu et al., 2019; Sekiya et al., 2013; Zhou et al., 1996). In this study, we revealed another important role for Nr4a factors in immune tolerance, i.e., regulation of peripheral Th and Treg differentiation at an early phase, at which Nr4a factors promote Treg cell differentiation, and repress Th1 and Th2 cell differentiation.

### Limitations of the study

In this study, we revealed that Nr4a factors play essential roles in regulating Th and Treg cell differentiation from naive T cells. However, since differentiation of each Th and Treg subset usually proceeds in a mutually repressive manner, the extent to which the observed abnormality in Th1, Th2, and Treg cell differentiation of Nr4a-TKO cells is affected by the differentiation of other cell subsets is uncertain, particularly *in vivo*. Related to the above issue, relative contribution of the defective Treg cell differentiation and the enhanced Th2 cell differentiation of Nr4a-TKO cells to the exacerbation of airway inflammation is still unknown. In addition, since all experiments were performed with mice and mice cells, relevance of the observation in this study to human beings awaits further investigation.

### Resource availability

#### Lead contact

Further information and requests for resources and data should be directed to the lead contact, Takashi Sekiya Ph.D.

Section of Immune Response Modification, Department of Immune Regulation, The Research Center for Hepatitis and Immunology, National Center for Global Health and Medicine, 1-7-1 Kohnodai, Ichikawa, Chiba 272-8516, Japan

Email: lb-sekiya@hospk.ncgm.go.jp

#### Materials availability

All unique reagents generated in this study are available from the lead contact without restriction.

#### Data and code availability

The accession numbers for the next generation sequencing data and microarray data are DNA Data Bank of Japan: DRA010571 and NCBI Gene Expression Omnibus: GSE153928, respectively. The DOI number for the raw data reported in this paper is Mendeley Data: https://doi.org/10.17632/8fnkj374kj.1

## Methods

All methods can be found in the accompanying Transparent methods supplemental file.

## Supplemental information

Document S1. Transparent methods and Figures S1–S4
