# Supplementary material for: Regulation of peripheral Th/Treg differentiation and suppression of airway inflammation by Nr4a transcription factors
Source: iScience. 2021 Feb 7;24(3):102166. doi: 10.1016/j.isci.2021.102166 (PMC7907427; doi:10.1016/j.isci.2021.102166)
Supplement: Document S1. Transparent methods and Figures S1–S4 [file mmc1.pdf]

iScience, Volume *24*

Supplemental information

Regulation of peripheral Th/Treg differentiation and suppression of airway inflammation by Nr4a transcription factors

Takashi Sekiya, Shizuko Kagawa, Katsunori Masaki, Koichi Fukunaga, Akihiko Yoshimura, and Satoshi Takaki

Supplemental Information

Supplemental Information contains Figures S1 to S4, the Transparent Methods, and Supplemental References.

Figure S1

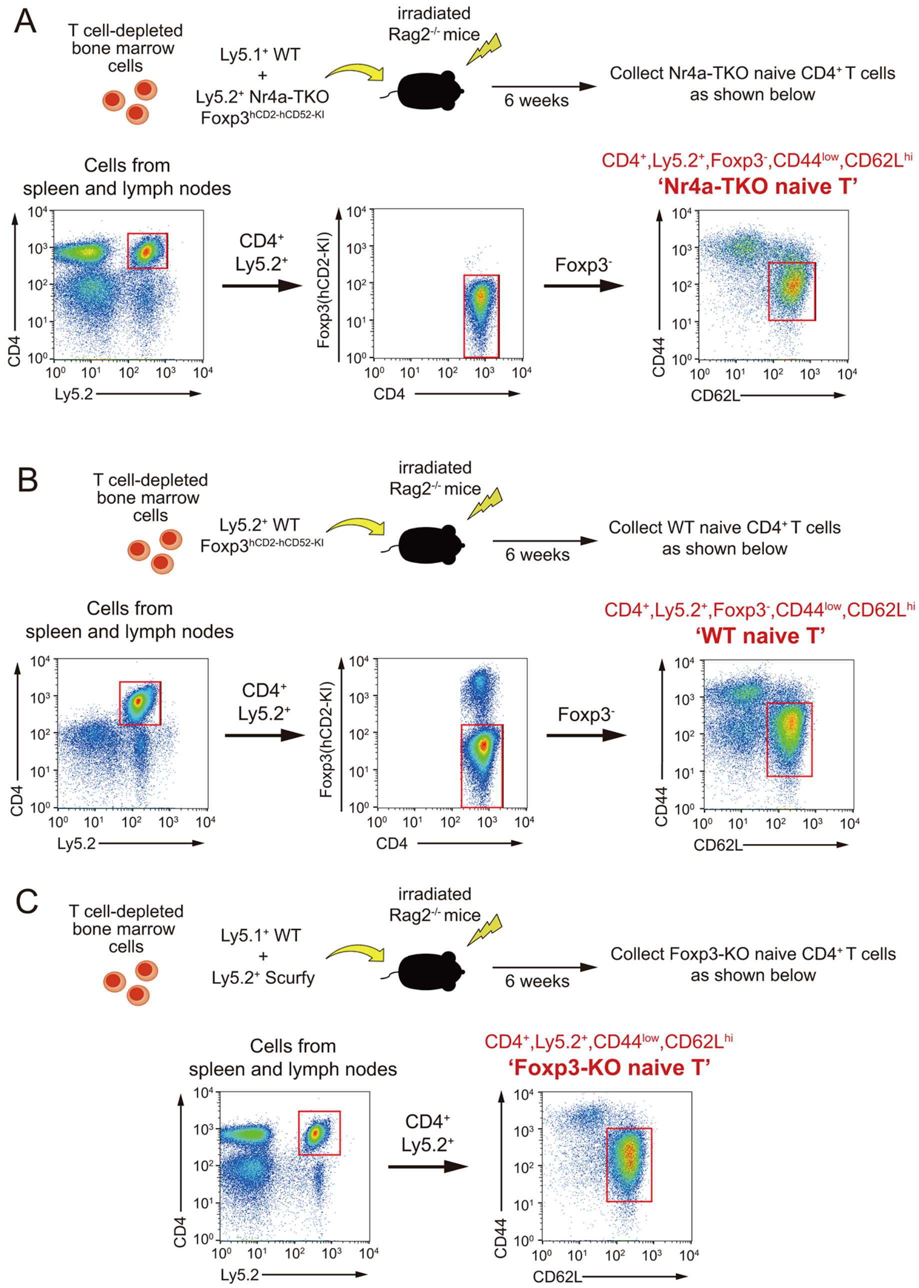

Figure S1 (Related to Figures 1-7): Strategy for the isolation of Nr4a-TKO naive T cells

(A) Gating strategy to obtain Nr4a-TKO naive T cells. Nr4a-TKO Treg cells were sorted as $CD4^{+}Ly5.2^{+}Foxp3^{-}CD44^{low}CD62L^{hi}$ cells from mixed bone marrow chimeras transferred

with wildtype (Ly5.1$^+$) and Nr4a-TKO Foxp3$^{hCD2hCD52\text{-}KI}$ (Ly5.2$^+$) cells. **(B)** Gating strategy to obtain wildtype naive T cells. Wildtype naive T cells were sorted as CD4$^+$Ly5.2$^+$Foxp3$^-$CD44^{low}CD62L^{hi} cells from bone marrow chimeras transferred with wildtype (Ly5.2$^+$) cells. **(C)** Gating strategy to obtain Foxp3-KO naive T cells. Foxp3-KO naive T cells were sorted as CD4$^+$Ly5.2$^+$CD44^{low}CD62L^{hi} cells from bone marrow chimeras transferred with wildtype (Ly5.1$^+$) and *scurfy* (Ly5.2$^+$) cells.

Figure S2

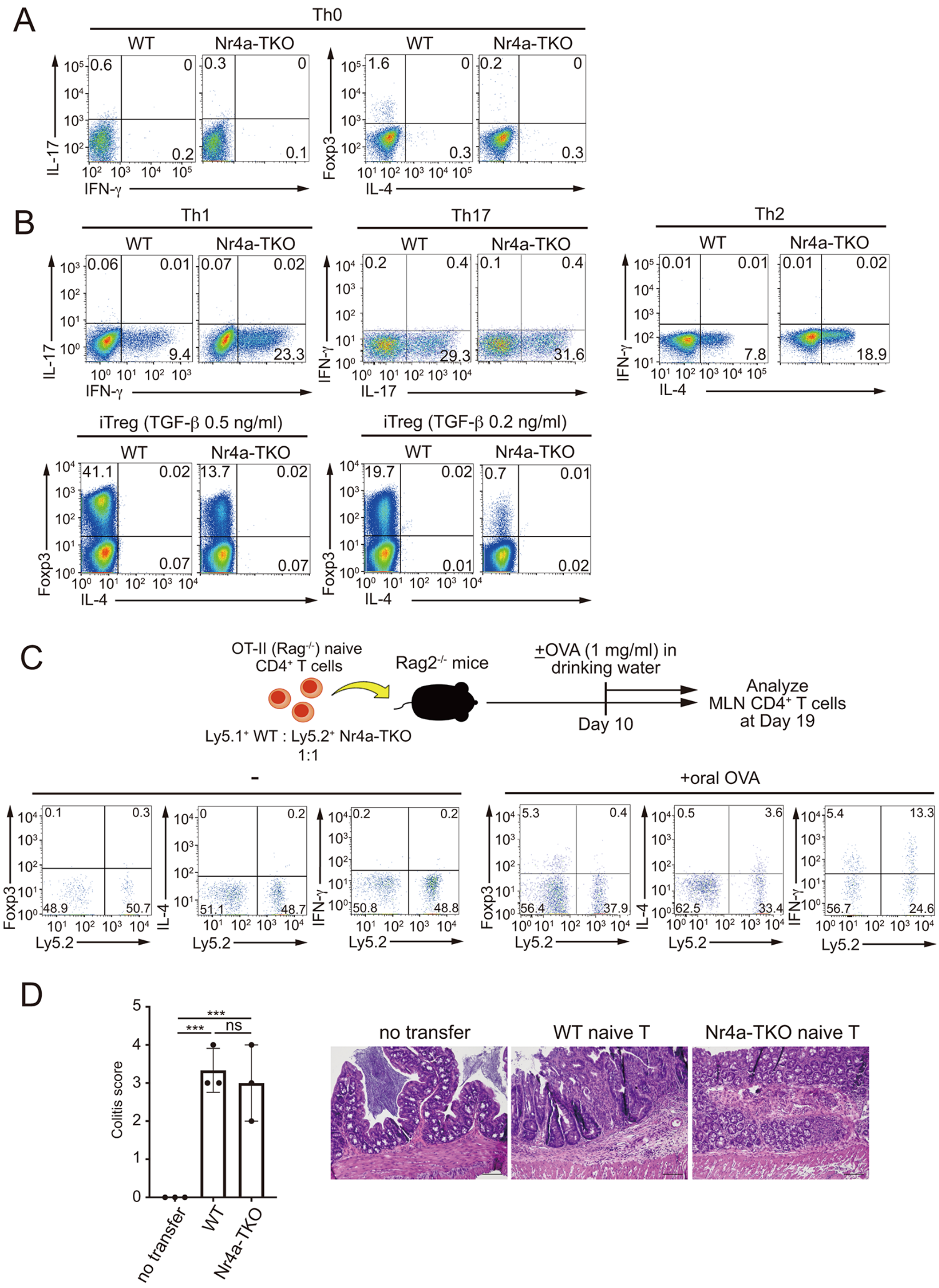
A
Th0
WT
Nr4a-TKO
IL-17
IFN-γ
Foxp3
IL-4
B
Th1
Th17
Th2
iTreg (TGF-β 0.5 ng/ml)
iTreg (TGF-β 0.2 ng/ml)
C
OT-II (Rag-/-) naive CD4+ T cells
Rag2-/- mice
±OVA (1 mg/ml) in drinking water
Day 10
Analyze MLN CD4+ T cells at Day 19
Ly5.1+ WT : Ly5.2+ Nr4a-TKO 1:1
-
+oral OVA
Ly5.2
D
Colitis score
***
ns
no transfer
WT
Nr4a-TKO
no transfer
WT naive T
Nr4a-TKO naive T

Figure S2 (Related to Figure 1 and Figure 2): Nr4a factors are important for induction of Treg cells and repression of Th1 and Th2 cell differentiation from naive T cells

(A) Flow cytometry profiles of wild type and Nr4a-TKO naive T cells cultured under Th0 condition, showing expression of the indicated proteins. Cells were analyzed 5 h after restimulation with PMA + ionomycin. Numbers in quadrants indicate percent cells in each. **(B)** Flow cytometry profiles of wildtype and Nr4a-TKO OT-II naive T cells cultured under the indicated conditions, showing expression of the indicated proteins. Cells cultured under Th1, Th2, and Th17 conditions were analyzed 5 h after restimulation with PMA + ionomycin. Numbers in quadrants indicate percent cells in each. **(C)** Top: A schematic of experiment performed. Bottom: Flow cytometry profiles of Ly5.2, Foxp3, IFN-γ, and IL-4 expression by total $CD3^+CD4^+$ cells from mesenteric lymph nodes (MLN) of $Rag2^{-/-}$ recipient mice treated as indicated. Numbers adjacent to outlined areas indicate percent cells in each. **(D)** Left: Histological score of colitis of $TCR\beta^{-/-}$ mice that had received wild type (n=3) or Nr4a-TKO naive T cells (n=3). Black dots represent individual values, and vertical bars indicate SD. Right: Hematoxylin and eosin staining of colon sections from recipient $TCR\beta^{-/-}$ mice at 30 days after transfer. Scale bars, 100 μm. ***$p < 0.005$; ns, not significant (one-way ANOVA with Bonferroni test).

Figure S3

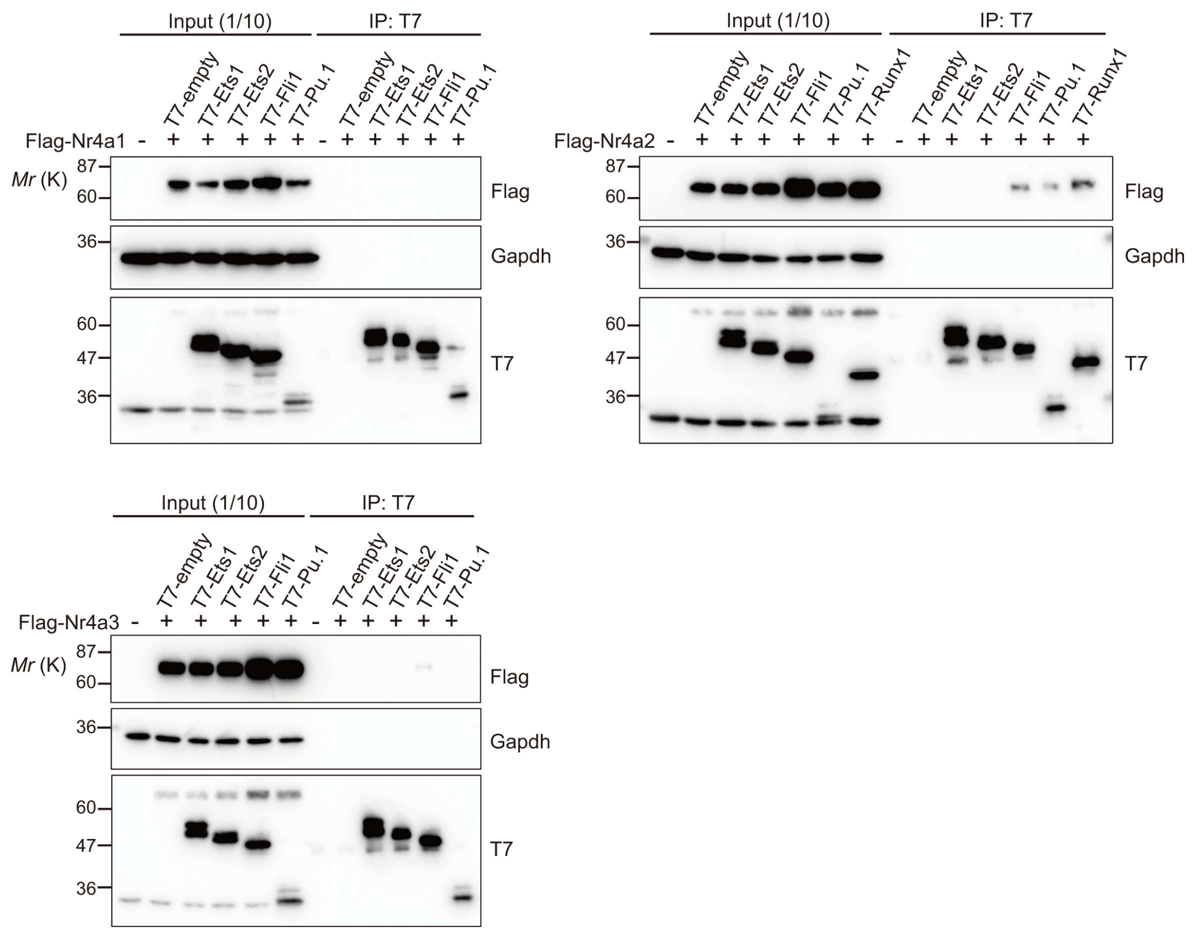

Figure S3 (Related to Figrue 4): Nr4a factors showed selective interaction with Ets factors

Co-immunoprecipitation of Flag-tagged Nr4a factors and T7-tagged Ets factors in 293T cell lysates with anti-T7-tag antibodies.

Figure S4

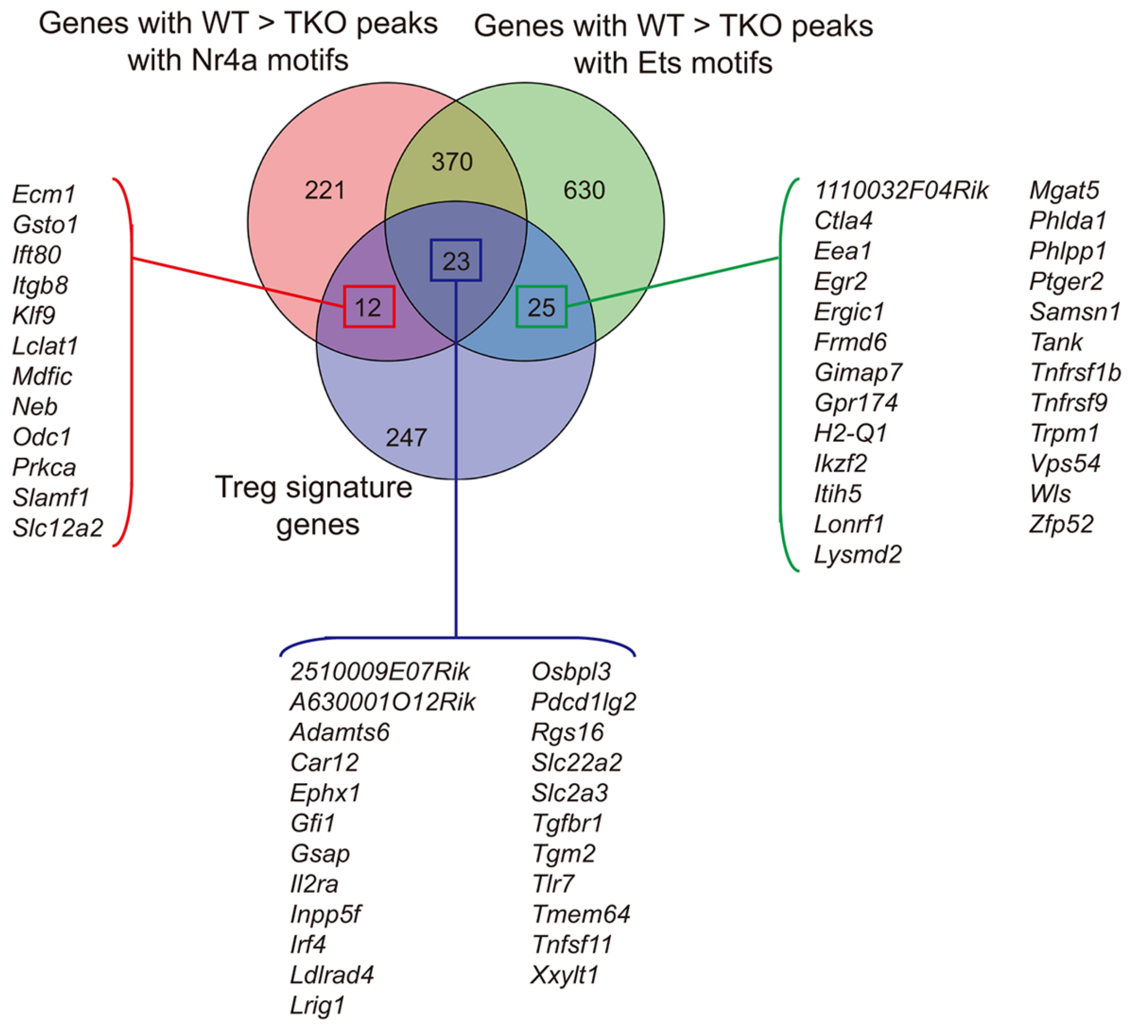

Figure S4 (Related to Figure 4): Nr4a and Ets factors regulate early responsive genes during iTreg cell differentiation

Venn diagram showing distribution of Treg signature genes with Nr4a or Ets motifs-containing ATAC-seq peaks that were more accessible in wild type iTreg cells at 3 h than in Nr4a-TKO iTreg cells at 3 h.

Transparent Methods

Mice

All mouse work was carried out in accordance with the guidelines for animal care approved by National Center for Global Health and Medicine and Keio University. Animals were maintained in specific pathogen-free conditions. 6-8 week old both male and female C57BL/6J *Nr4a1-floxed* and *Nr4a2-floxed* mice (Kadkhodaei et al., 2009, from Dr. H. Ichinose, Tokyo Institute of Technology, Japan), C57BL/6J *Nr4a3*$^{-/-}$ mice (TF0937, Taconic farm), C57BL/6J TCRβ$^{-/-}$ (002122, Jackson Laboratories), C57BL/6J *Rag2*$^{-/-}$ (008449, Jackson Laboratories), C57BL/6J *Foxp3*$^{hCD2\text{-}hCD52\text{-}KI}$ mice (Komatsu et al., 2009, from Dr. S. Hori, RIKEN, Japan), and male C57BL/6J *Scurfy* mice (Jackson Laboratories) were bred in NCGM's experimental animal care facility. Breeding animals were fed ''CE2'' chow (CLEA, Japan). Experiments were performed with age-matched cohorts. We observed no significant differences in autoimmune phenotypes in male versus female Nr4a-TKO mice. We also observed no significant differences in disease phenotypes between male and female mice in utilizing TCRβ$^{-/-}$ and Rag2$^{-/-}$ mice as recipients of naive T cells. Foxp3-KO (Scurfy) mice were all male, as those mice were obtained only by the mating between Scurfy/+ female mice and +/Y male mice. ATAC-seq was performed with cells only from male mice, as Foxp3 is on the X chromosome, thus one of the two alleles are inevitably methylated in female cells.

Antibodies

The monoclonal antibody against Nr4a1 (H1648), Nr4a2 (N1404), and Nr4a3 (H7833) were purchased from Perseus Proteomics. Antibodies to Gapdh (3H12) was purchased from MBL. Phycocerythrin (PE)-, PerCP-Cy5.5-, Allophycocyanin (APC)-, or APC-Cy7-conjugated monoclonal antibodies for CD4 (L3T4), hCD2 (LFA-2), Foxp3 (FJK-16s), IL-4 (11B11), IFN-γ (KMG1.2), Thy1.1 (HIS51), and IL-17a (eBio17B7) antibodies were purchased from eBioscience or BioLegend. Anti-Nr4a1 antisera used in ChIP-seq experiments were raised by immunizing rabbits with recombinant Nr4a1 protein (aa2-266) generated in E. coli.

ELISA

For analysis of immunoglobulin concentration, sera or BAL were subjected to Mouse Ig ELISA Quantitation Kit (Bethyl), according to the manufacturer's protocol. For quantification of OVA specific IgE in BAL, plates were coated overnight at 4 ^{o}C with 2 μg Ovalbumin per well, instead of coating the plates with capture antibody for IgE.

Histopathological examination

Tissues were fixed in 4% formalin. Fixed tissues were embedded in paraffin, and were then cut into 5 μm sections. Samples were stained with periodic acid-Schiff (PAS) staining to identify the degree of mucus secretion in the lung tissue, as described previously (Tamiya et

al., 2013).

Isolation of naïve T cells

Nr4a-TKO and Foxp3-KO naïve $CD4^+$ T cells ($Ly5.2^+CD4^+CD62L^{hi}CD44^{low}Foxp3^-$) were sorted from mixed bone marrow chimera given wild-type (Ly5.1) and knockout (Ly5.2) bone marrow cells (T cell depleted) at 1:1 ratio, six weeks after bone marrow transplantation. Wild type naïve $CD4^+$ T cells ($Ly5.2^+CD4^+CD62L^{hi}CD44^{low}Foxp3^-$) were sorted from bone marrow chimera given wild type bone marrow cells (T cell depleted), six weeks after bone marrow transplantation. In the bone marrow transplantation, bone marrow cells were intravenously transferred to *Rag2*$^{-/-}$ recipients irradiated at 8.5 Gy. Wild type and Nr4a-TKO OT-II naive T cells were isolated from OT-II-$Rag2^{-/-}Foxp3^{hCD2\text{-}hCD52\text{-}KI}$ mice and Nr4a-TKO-OT-II-$Rag2^{-/-}Foxp3^{hCD2\text{-}hCD52\text{-}KI}$ mice respectively, by sorting $CD4^+CD62L^{hi}CD44^{low}CD25^{low}Foxp3^-$ populations.

In vitro culture of primary naive $CD4^+$ T cells

Primary naïve $CD4^+$ T cells were cultured in RPMI1640 medium supplemented with plate-coated anti-CD3ε (clone 2C11, 2 μg/ml for Th1, Th17, and iTreg conditions, and 0.5 μg/ml for Th2 conditon), anti-CD28 (clone 57.31, 0.5 μg/ml) antibodies, 2-Mercaptoethanol (Invitrogen, 55 μM), 10% fetal bovine serum, and further supplemented with the following for each skewing condition: for Th0: anti-IFN-γ (clone R4-6A2, 1 μg/ml), anti-IL-4 (clone

11B11, 1 μg/ml) antibodies, and IL-2 (10 ng/ml, PeproTech); for iTreg: anti-IFN-γ (clone R4-6A2, 1 μg/ml), anti-IL-4 (clone 11B11, 1 μg/ml) antibodies, IL-2 (10 ng/ml, PeproTech) and recombinant human TGF-β1 (0.2 or 0.5 ng/ml, BioLegend); for Th1: anti-IL-4 (clone 11B11, 1 μg/ml), IL-2 (10 ng/ml, PeproTech), IL-12 (20 ng/ml, PeproTech); for Th2: anti-IFN-γ (clone R4-6A2, 1 μg/ml), IL-2 (10 ng/ml, PeproTech), IL-4 (20 ng/ml, BioLegend); and for Th17: anti-IL-2 (clone JES6-1A12, 1 μg/ml), anti-IFN-γ (1 μg/ml), anti-IL-4 (clone 11B11, 1 μg/ml) antibodies, human IL-6 (20 ng/ml, R&D Systems), and recombinant human TGF-β1 (1 ng/ml, BioLegend).

Adoptive transfer of naive $CD4^+$ T cells into $TCR\beta^{-/-}$ recipient mice

Sorted naïve $CD4^+$ T cells were intravenously transferred into 6- to 8-week-old $TCR\beta^{-/-}$ mice (which do not have αβT cells, but have B cells). 30 days after transfer, the sera, BAL, and organs were collected for analysis. For the analysis of colitis, histological grades were assigned in a blinded manner, on a scale of 0 to 5 where a grade of 0 was given when there were no changes observed. Grade 1 exhibits minimal scattered mucosal inflammatory cell infiltrates, with or without minimal epithelial hyperplasia; grade 2, mild scattered to diffuse inflammatory cell infiltrates, sometimes extending into the submucosa and associated with erosions, with mild to moderate epithelial hyperplasia and mild to moderate mucin depletion from goblet cells; grade 3, moderate inflammatory cell infiltrates that were sometimes transmural, with moderate to severe epithelial hyperplasia and mucin depletion; grade 4,

marked inflammatory cell infiltrates that were often transmural and associated with crypt abscesses and occasional ulceration, with marked epithelial hyperplasia, mucin depletion; and grade 5, marked transmural inflammation with severe ulceration and loss of intestinal glands.

In vivo antigen stimulation of Rag2$^{-/-}$ OT-II TCR-Tg naive T cells

Sorted naïve CD4^{+} T cells from Ly5.1^{+} wild type Rag2$^{-/-}$ OT-II TCR-Tg mice and Ly5.2^{+} Nr4a-TKO Rag2$^{-/-}$ OT-II TCR-Tg mice, both 1 x 10^{5} cells, were intravenously co-transferred into 6- to 8-week-old Rag2$^{-/-}$ mice (set as day 0). For stimulating cells under a tolerizing condition, mice were orally administrated with OVA (A5503, Sigma) in drinking water (1%) from day 10 to day 19. For stimulating cells under an inflammatory condition, mice were intraperitoneally injected with 100 μg Ovalbumin in 1 mg of Imject™ Alum Adjuvant (Thermo Fisher Scientific), at day 10. At day 19, cells were collected from mesenteric lymph nodes and analyzed by flow cytometry.

Assessment of airway hyperresponsiveness to methacholine

Serial dilutions of acetyl β-methacholine in sterile normal saline were prepared fresh daily. Untreated TCRβ$^{-/-}$ mice and TCRβ$^{-/-}$ recipient mice of wildtype and Nr4a-TKO naive T cells at 30 d after transfer were mechanically ventilated on a computer-controlled piston ventilator, flexiVent system (SCIREQ, Montreal, Canada). Mice were exposed to increasing doses of

methacholine (0, 6, 12, 25, 50, and 100 mg/ml). 10 recordings of total lung resistance were generated at each methacholine dose. After each methacholine challenge, the airway resistance was measured every 15 s during tidal breathing, and the 3rd or 4th measurement whichever was higher was used as the value of bronchoconstrictor response to each individual methacholine concentration. Overall group mean values were then calculated at each methacholine dose.

Western blotting

Proteins were dissolved in SDS-PAGE sample buffer, then separated by SDS-PAGE and electro-transferred onto an Immobilon-P PDVF membrane (Millipore). Membranes were hybridized with the following antibodies: anti-GAPDH mAb (3H12, MBL), anti-Nr4a1 mAb (H1648), anti-Nr4a2 mAb (N1404), anti-Nr4a3 mAb (H7833). After being hybridized with HRP-conjugated secondary antibodies, membranes were visualized using an ImmunoStar-LD detection system (WAKO).

Microarray analysis of mRNA

Total RNA was extracted with RNAiso PLUS (TAKARA). Samples were further cleaned using an NucleoSpin RNA Clean-up XS (MACHEREY-NAGEL), labeled with Cyanine 3-CTP using a Low Input Quick Amp Labeling Kit (Agilent Technologies), and hybridized to a 8x60K SurePrint G3 Mouse GE microarray kit (Agilent Technologies). Expression values

for each probe set were calculated using the RMA method with GeneSpring GX 12.6.1 software (Agilent Technologies). Gene set enrichment analysis (GSEA) was run with GSEA_4.0.3, on wild type and Nr4a-TKO iTreg 24 h microarray data, which were formatted into a single gct file. This gct file was then screened against a GMT file that contains gene sets from MSigDB C7: immunologic signatures.

ATAC-seq

ATAC-seq was performed using Nextera DNA Library Preparation Kit (illumina). $4x10^5$ cells (male origin) were suspended in 200 μl Hypotonic buffer (20 mM Tris pH 7.5, 10 mM NaCl, 3 mM), and incubated for 15 min on ice. Then, 10 μl 10% NP-40 was added to the sample and vortexed for 10 sec at maximum speed. Cells were pelleted by centrifugation at 6,000 rpm for 5 min, then suspended in 40 μl 1x Tagmentation buffer. Samples were added with 2 μl Tagment DNA Enzyme 1, and incubated for 30 min at 37 ^{o}C. Then, samples were cleaned up with FastGene Gel/PCR clean up kit (Nippon Genetics), and eluted with 20 μl elution buffer. 5 μl of the eluted samples were PCR amplified with index 1 (i7) and index 2 (i5) adapter primers, using PCR Primer Cocktail which was attached to Nextera DNA Library Preparation Kit. 150 bp paired end next generation sequencing was performed with HiSeq X Ten (illumina). Generated sequencing reads in FASTQ format were mapped to mm10 reference genome using bowtie2. Reads mapped to the "black list" regions were removed with intersectBed command, inputting the bed file which denotes the black list region

(mm10-blacklist.v2.bed.gz). Peak calling was performed with MACS14, with default parameters. Differentially accessible genomic regions among the samples were detected with an R package DiffBind. Generated bed files were employed to obtain sequences spanning the corresponding genomic regions, by "blastdbcmd" command against mm10 reference genome, that was formatted with "makeblastdb" command. Then, the generated multi-fasta file was analyzed with MEME to obtain motifs which were enriched in the input genomic sequences. Gene annotation of the intended genomic regions were performed with HOMER's "annotatePeaks.pl" command.

ChIP-seq

1.5×10^7 wildtype iTreg cells at 3 h (male origin) were fixed with 1 ml of 1% formaldehyde for 10 min with occasional swirling at room temperature. Crosslinking was stopped by addition of glycine to 125 mM. Cells were then lysed in 100 μl of lysis buffer (20 mM Tris (pH 7.5), 5 mM EDTA, and 0.5 % SDS for 10 min on ice. Genomic fragments were sonicated to a mean size of approximately 200 bp using an Acoustic Solubilizer (Covaris) with a 200 bp shearing protocol. After insoluble material was removed by centrifugation at 14,000 rpm for 10 min, 5 μl of the supernatants were taken as input, and the remainder was diluted five-fold with dilution buffer (1% Triton X-100, 1 mM EDTA, 150 mM NaCl, and 20 mM Tris (pH 8.0), supplemented with protease inhibitor cocktail). Then, chromatin samples were pre-cleared for 1 h at 4 ^{o}C with 5 μl of Protein A/G PLUS-Agarose (Santacruz), which had been

blocked with 500 μg/ml BSA. Immunoprecipitation of the pre-cleared samples were performed with 5 μl of in-house generated anti-mouse Nr4a1 antisera (described in the "Antibodies" section) or with 5 μl of pre-immune sera, for 4 h at 4°C with rotation. Complexes were recovered by incubation with 10 μl of Protein A/G PLUS-Agarose, which had been blocked with 500 μg/ml BSA. Precipitates were washed serially with 500 μl RIPA buffer (50 mM Tris pH 8.0, 150 mM NaCl, 0.1% SDS, 0.5% Na-deoxycholate, 1% NP-40, 1 mM EDTA), 500 μl high salt buffer (50 mM Tris (pH 8.0), 500 mM NaCl, 0.1% SDS, 0.5% Na-deoxycholate, 1% NP-40, 1 mM EDTA), 500 μl LiCl buffer (50 mM Tris (pH 8.0), 1 mM EDTA, 250 mM LiCl, 1% NP-40, and 0.5% Na-deoxycholate), and then twice with 500 μl TE. Chromatin samples were eluted from the beads three times with 70 μl elution buffer (1% SDS and 0.1 M $NaHCO_3$) for 30 min at room temperature with constant agitation. Then, crosslinking was reversed by overnight incubation at 65°C. After the reversal of crosslinking, proteinase K was added to the samples at 0.2 mg/ml, and the samples incubated for 5 h at 56°C. After phenol-chloroform extraction, the aqueous phase was ethanol-precipitated and dissolved in 50 μl TE buffer. Libraries for next generation sequencing was prepared from 10 ng of the immunoprecipitated DNA fragments, using TruSeq DNA Sample prep Kit. 150 bp paired end next generation sequencing was performed with NovaSeq6000 (illumina). Generated sequencing reads in FASTQ format were mapped to mm10 reference genome using bowtie2. Reads mapped to the "black list" regions were removed with intersectBed command, inputting the bed file which denotes the black list region (mm10-

blacklist.v2.bed.gz). Peak calling was performed with HOMER's "FindPeaks.pl" command, with -style factor -o auto options. Enriched motifs in the mapped genomic regions were detected with HOMER's "findMotifsGenome.pl" command, with default parameters. Joint analysis of ChIP-seq and ATAC-seq results were performed using ngs.plot.r algorithm, with following commands: ngs.plot.r -G mm10 -R bed -C [ChIP-seq bam file] -E [ATAC-seq summit bed file] -L 1000 -FL 150.

Co-immunoprecipitation

For immunoprecipitation, cells were lysed in lysis buffer (140 mM NaCl, 1% Triton X-100, 1 mM EDTA, 10 mM Tris [pH 8.0], supplemented with protease inhibitor cocktail (Nacalai Tesque) for 1 h at 4°C. Cell lysates were incubated with 1 μg of anti-T7 rabbit polyclonal antibodies (PM022, MBL) for 4 h at 4°C, then incubated with 15 μl protein G-Sepharose (GE Healthcare). Immunoprecipitates were washed five times with wash buffer (140 mM NaCl, 0.1% Triton X-100, 1 mM EDTA, 10 mM Tris [pH 8.0]). Proteins were dissolved in SDS-PAGE sample buffer, then separated by SDS-PAGE and electrotransferred to an Immobilon-P PDVF membrane (Millipore, Billerica, MA, USA). Membranes were hybridized with anti-T7 (cat# 69522, Merck), anti-Flag (clone M2, SIGMA), and anti-Gapdh (M171-3, MBL) mouse monoclonal antibodies, then visualized using an ImmunoStar® Zeta (FUJIFILM Wako Chemicals).

Luciferase assay

Batf3-Luc reporter plasmid was transfected into Jurkat cells along with expression plasmids for Batf, JunB, Irf4, Nr4a1, Nr4a2, and Nr4a3, by electroporation with Neon Transfection System (Thermo Fisher Scientific). Total amounts of plasmids were adjusted with an empty plasmid. 24 h post-transfection, cells were harvested, and luciferase activities were measured using the Dual-Luciferase reporter assay system (Promega) according to the manufacturer's protocol. Activities of Batf3-Luc reporter were normalized against Renilla luciferase activities from co-transfected pRL-tk plasmids.

Mouse model of allergic airway inflammation with oral tolerance

TCRβ$^{-/-}$ mice were intravenously transferred with 1x10^{5} wild type or Nr4a-TKO OT-II TCR-Tg (Rag2$^{-/-}$ background) naive T cells (set as day 0). 19 d and 26 d after transfer, mice were intraperitoneally injected with 100 μg OVA in 1 mg of Imject^{TM} Alum Adjuvant (Thermo Fisher Scientific). From seven days after the second immunization, mice were nasally challenged with OVA (5% in PBS) for 15 min by a nebulizer, for 5 five consecutive days. Cohorts of mice were orally administrated with OVA in drinking water (1%) for 7 days, from day 10 to day 17. One day after the final nasal challenge with OVA, mice were sacrificed, and the samples were collected from spleen, lung, and BAL for analysis.

Construction of Nr4a2-ER-Tg OT-II naive T cells

Bone marrow cells were collected from the tibia and femur of Rag2$^{-/-}$ OT-II TCR-Tg mice (Foxp3$^{hCD2\text{-}hCD52\text{-}KI}$ background), 5 d after intraperitoneal injection of 5 mg 5-fluorouracil (Sigma) in PBS. Cells were cultured for 4 d at a density of 1×10^6 cells/ml with mouse IL-3 (20 ng/ml), mouse IL-6 (50 ng/ml) and mouse SCF (50 ng/ml; all obtained from PeproTech), in DMEM (low-glucose) containing 10% FBS and 2-Mercaptoethanol (Invitrogen, 55 μM). At 48 and 72 h of culture, bone marrow cells were transduced with Nr4a2-ER–expressing retrovirus or control retrovirus, that express only the Thy1.1 reporter, by centrifugation at 2,500 r.p.m. for 2 h at 35 °C, in a solution containing polybrene (2 μg/ml). After transduction, the retroviral supernatant was removed and replaced with same fresh growth media. At day 4, retrovirally transduced bone marrow cells ($1x10^6$ cells/mouse) were intravenously transferred into Rag2$^{-/-}$ mice irradiated at 9.5 Gy. Six weeks later, mice were sacrificed and naive T cells that express the transgenes ($CD4^+CD25^{low}CD62L^{hi}CD44^{low}Foxp3^-Thy1.1^+$) were sorted.

Pharmacological activation of Nr4a2-ER chimeric molecule in mouse model of allergic airway inflammation.

1×10^5 Nr4a2-ER-Tg or non-Tg (express only Thy1.1 reporter) OT-II naive T cells were intravenously transferred into TCRβ$^{-/-}$ mice (set as day 0). From day 8, mice were intraperitoneally administered 0.3 mg tamoxifen (MP Biomedicals) dissolved in corn oil (Sigma), for 5 consecutive days. At day 10 and 17, mice were intraperitoneally injected with

100 μg OVA (Sigma) in 1 mg of Imject™ Alum Adjuvant (Thermo Fisher Scientific). From day 21, mice were nasally challenged with OVA (5% in PBS) by nebulizer for 15 min, for 5 five consecutive days. One day after final nasal challenge with OVA, mice were sacrificed, and samples were collected from spleen, lung, and BAL for analysis.

Statistical analysis

p values were calculated with Graphpad Prism software and R. *p* values of less than 0.05 were considered significant. All error bars in graphs represent SEM calculated at least three replicates. Data were assessed for normal distribution and plotted in the figures as mean ± SD. No samples or animals were excluded from the analyses. Differences between two treatment groups were assessed using two-tailed, unpaired Student *t* test with Welch's correction. Comparisons for more than two groups with only one variable were assessed using one-way ANOVA with an appropriate post hoc tests (e.g., Bonferroni's test and Kruskal-Wallis test). Statistical tests for comparisons of ≥ two groups with two variables were performed with two-way ANOVA with Sidak test.

Supplemental References

Kadkhodaei, B., Ito, T., Joodmardi, E., Mattsson, B., Rouillard, C., Carta, M., Muramatsu, S., Sumi-Ichinose, C., Nomura, T., Metzger, D., *et al.* (2009). Nurr1 is required for maintenance of maturing and adult midbrain dopamine neurons. J Neurosci *29*, 15923-15932.

Komatsu, N., Mariotti-Ferrandiz, M.E., Wang, Y., Malissen, B., Waldmann, H., and Hori, S. (2009). Heterogeneity of natural Foxp3+ T cells: a committed regulatory T-cell lineage and an uncommitted minor population retaining plasticity. Proc Natl Acad Sci U S A *106*, 1903-1908.

Tamiya, T., Ichiyama, K., Kotani, H., Fukaya, T., Sekiya, T., Shichita, T., Honma, K., Yui, K., Matsuyama, T., Nakao, T., *et al.* (2013). Smad2/3 and IRF4 play a cooperative role in IL-9-producing T cell induction. J Immunol *191*, 2360-2371.
